# Bio-high entropy alloys: Progress, challenges, and opportunities

**DOI:** 10.3389/fbioe.2022.977282

**Published:** 2022-09-08

**Authors:** Junyi Feng, Yujin Tang, Jia Liu, Peilei Zhang, Changxi Liu, Liqiang Wang

**Affiliations:** ^1^ School of Materials Engineering, Shanghai University of Engineering Science, Shanghai, China; ^2^ Affiliated Hospital of Youjiang Medical University for Nationalities, Baise, China; ^3^ State Key Laboratory of Metal Matrix Composites, School of Materials Science and Engineering, Shanghai Jiao Tong University, Shanghai, China

**Keywords:** biological high-entrogy, compositon design, mechanical properties, implant, biocompatibility

## Abstract

With the continuous progress and development in biomedicine, metallic biomedical materials have attracted significant attention from researchers. Due to the low compatibility of traditional metal implant materials with the human body, it is urgent to develop new biomaterials with excellent mechanical properties and appropriate biocompatibility to solve the adverse reactions caused by long-term implantation. High entropy alloys (HEAs) are nearly equimolar alloys of five or more elements, with huge compositional design space and excellent mechanical properties. In contrast, biological high-entropy alloys (Bio-HEAs) are expected to be a new bio-alloy for biomedicine due to their excellent biocompatibility and tunable mechanical properties. This review summarizes the composition system of Bio-HEAs in recent years, introduces their biocompatibility and mechanical properties of human bone adaptation, and finally puts forward the following suggestions for the development direction of Bio-HEAs: to improve the theory and simulation studies of Bio-HEAs composition design, to quantify the influence of composition, process, post-treatment on the performance of Bio-HEAs, to focus on the loss of Bio-HEAs under actual service conditions, and it is hoped that the clinical application of the new medical alloy Bio-HEAs can be realized as soon as possible.

## Introduction

As one of the material foundations of human production and life, metal materials have always played an essential role in the development history of human civilization. In recent decades, with the continuous development of science and technology, people have put forward higher and higher requirements for the comprehensive properties of metal materials. People have been changing the properties of materials by adding relatively small amounts of secondary elements to the primary elements. For example, C and Cr elements are added to steel to improve strength and corrosion resistance, and Al-Mn and Al-Mg alloys formed by adding Mn and Mg to aluminum have good corrosion resistance and plasticity (Zhang et al., 2008; Serda, 2013; Liu et al., 2021; Zhou et al., 2022). However, such a primary-element approach dramatically limits the total number of possible element combinations and, therefore, the total number of alloys, most of which have been identified and exploited. New approaches are needed if the compositional space for exploration is significantly enlarged. To obtain alloy materials with better properties, in the 1990s, researchers got alloys with high mixing entropy by adding alloy components (Peker and Johnson, 1993; Choi-Yim and Johnson, 1997). In 2004, Ye et al. (Yeh et al., 2004) prepared multi-principal composition alloys with equal or nearly equal molar ratios, and named such multi-principal alloys as high entropy alloys (HEAs) for the first time. And unlike conventional alloys, the properties of HEAs are jointly influenced by multiple constituent elements. High entropy alloys have advantages not found in conventional alloys, such as high strength, high-temperature resistance, corrosion resistance, etc. (Yeh, 2006, 2013; Senkov et al., 2010, 2011; Miracle et al., 2014; Zhang et al., 2014, 2018; George et al., 2019; Xin et al., 2020).

**FIGURE 1:**
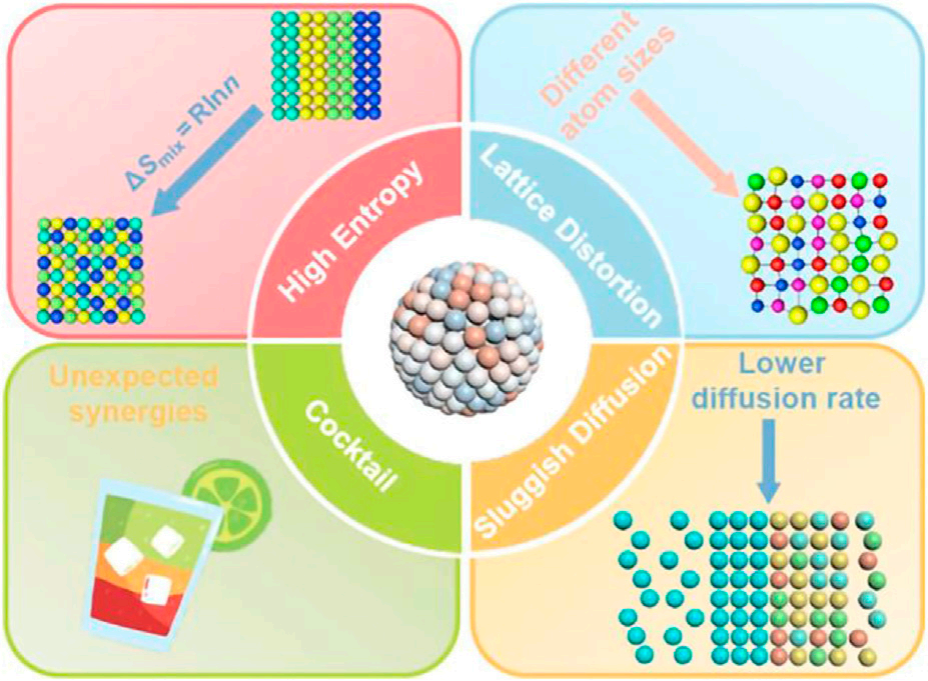
Schematic illustration of the properties and characteristics of HEAs (Xin et al., 2020).

At present, high-entropy alloys refer to a class of alloys composed of five or more elements, and the atoms of each component are smelted and alloyed according to an equal atomic ratio or close to an equal atomic ratio and have high mixing entropy and solid solution formation tendency (Schopphoven et al., 2016; Liang et al., 2022). Scholars have conducted a lot of research on HEA and have concluded four core effects: high entropy effect, sluggish diffusion, lattice distortion, and cocktail effect, as shown in Figure 1 (Xin et al., 2020). Among them, high lattice distortion and high mixing entropy will lead to a large degree of atomic disorder in the alloy. This allows HEA to have low Gibbs free energy, which significantly improves the stability of the single solid solution phase and inhibits the formation of intermetallic compounds (Wang B. et al., 2018; Huo et al., 2018; Nong et al., 2018). Structural “lattice distortion effect,” that is, the difference in atomic size among various elements, can cause severe lattice distortion, which is considered to be the primary reason for the high strength of high-entropy alloys and has an essential impact on the morphology and movement of dislocation lines (Ma Y. et al., 2020; Xie et al., 2020). The cocktail effect refers to the fact that HEA is an alloy formed by mixing multiple elements and will exhibit properties that a single pure metal element does not have (Lin C. L. et al., 2021). These properties give HEA a more comprehensive range of applications (Wang et al., 2019; Wei et al., 2021; Jiang et al., 2022a, 2022b; Cheng et al., 2022).

Currently, potential applications for HEAs include corrosion-resistant materials, nuclear materials, molds, and biomedicine (George et al., 2020; Soto et al., 2020). Among these, HEAs have great potential in the biomedical field, and biomaterials science continues to be at the forefront of research and innovation in clinical and biomedical applications as medical technology advances and the needs of the population increase. An illustrative example of the significant impact is that degenerative diseases of the bones and joints, such as osteoporosis, affect many people worldwide, mainly middle-aged and postmenopausal women (Cohen et al., 2022; Iki et al., 2022; Mattia et al., 2022). Growing clinical demand for reconstructive joint replacements is prompting researchers to develop implants with better function, biocompatibility, and improved clinical outcomes. Alloys have been used as bone implants for many years. Among them, stainless steel, cobalt-based alloys (CoCrMo), and titanium and their alloys are widely used for their good biocompatibility, sufficient mechanical strength, and excellent corrosion resistance (Gross et al., 2020; Shipilova et al., 2020). However, implants made from these materials are usually much stiffer than natural bone, leading to stress shielding - a significant source of bone resorption and eventual failure of such implants. The human skeleton can be divided into dense bone (cortical bone) and trabecular bone (cancellous bone). Dense bone is almost solid, while the porosity of trabecular bone varies between 50 and 90%, and the mechanical properties of the bone vary significantly with age, anatomical location, and bone mass (Lei et al., 2013; Al-Hafidh et al., 2020; Gaffuri et al., 2021). Such complex mechanical property modulation is difficult to achieve with a single principal element alloy, which requires substantial elemental modulation by HEA to meet specific bone-implant needs. Such complex mechanical property modulation is difficult to achieve with a single primary element alloy, which requires significant elemental modulation of HEA to meet specific bone-implant needs.

We call this HEA with biomedical application potential Biological High Entropy Alloy (Bio-HEAs) [41], and many scholars have already researched the related direction. Metal biomaterials must be composed of raw materials with good biocompatibility, such as non-toxic and non-allergenic materials (Meloni et al., 2019; Kazemi et al., 2020; Spataru et al., 2021). The selection of constituent elements is particularly critical for Bio-HEA. Bio-HEA is mainly composed of Ti, Zr, Hf, Nb, Ta, V, Mo, and W. These elements do not cause side effects to the tissue cells at the implantation site or are within the safe range of side effects to the body throughout the service phase. High-entropy alloys have become one of the most promising medical metal materials in recent years due to their biological safety, high strength, high corrosion resistance, high wear resistance, and ease of forming simple objects. Since high entropy alloys are tunable in terms of properties, the desired properties can be obtained by changing the type or content of the elements in the HEAs, which gives the HEAs a broader scope of application. In the field of biomedicine, high-entropy alloys have a similar hardness to the bone, high specific strength, good corrosion, and wear resistance, and these characteristics are in line with the typical attributes of biomedical metal materials, which means that there is a good potential for its application in the medical health field.

**FIGURE 2:**
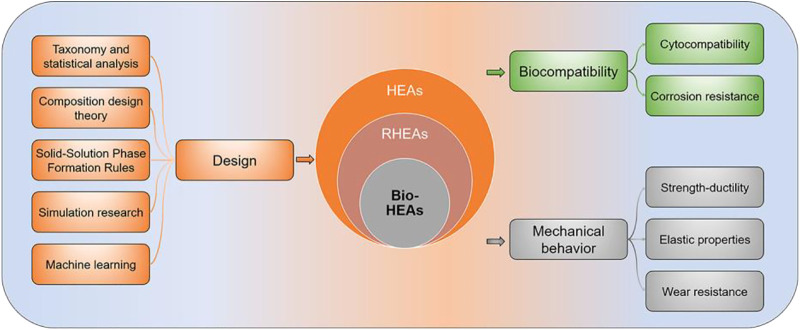
A mind map to explore the design theory, biocompatibility, and mechanical properties of Bio-HEAs in this paper.

Figure 2 shows the structure and main content of the paper. The second section reviews the composition design theory of Bio-HEAs. It presents the progress of research on developing Bio-HEAs compositions based on the first principles of density functional theory (DFT) simulations calculation. The third section discusses the biocompatibility of the designed and developed Bio-HEAs, including cytocompatibility and corrosion resistance. The fourth section compares the mechanical properties of Bio-HEAs with human bone and commonly used bio-alloys, including strength-ductility, elastic properties, and wear resistance. The fifth section discusses the future development prospects of Bio-HEAs and explores the possibility of high-entropy alloys in biomedicine.

## Component design theory and simulation studies

The proposal of the concept of the high-entropy alloy not only improves the freedom of alloy material composition but also dramatically increases the difficulty of its composition exploration and performance optimization. Selecting alloying elements and the appropriate proportions are particularly important to obtain high-entropy alloys with specific phase structures or properties (Pradeep et al., 2015; Takeuchi et al., 2015; Tian et al., 2015; Ye et al., 2015). The core problems involved in the current high entropy alloys research can often be summarized as the problem of alloy composition design and property optimization (LaRosa et al., 2019; George et al., 2020; Soto et al., 2020).

As one of the members of high-entropy alloys, Bio-HEA has the same component design concept as high-entropy alloys. Due to the large number of optional components of HEAs and the high content of each component, the complex physical properties and chemical synergism between various alloying elements will ultimately significantly affect the mechanical properties and microstructure of high-entropy alloys (Cann et al., 2021; Ostovari Moghaddam et al., 2021). This chapter summarizes the research on Bio-HEAs in recent years as a composition system. The combined effects of all the components are considered from composition design theory. The phase formation laws and mechanisms are discussed through solid-solution phase formation rules. The Bio-HEAs alloy properties are predicted by simulation, such as first principles and molecular dynamics. Machine learning is introduced to provide a reference database for the composition design guidelines of new high-entropy alloys.

### Taxonomy and statistical analysis

**FIGURE 3:**
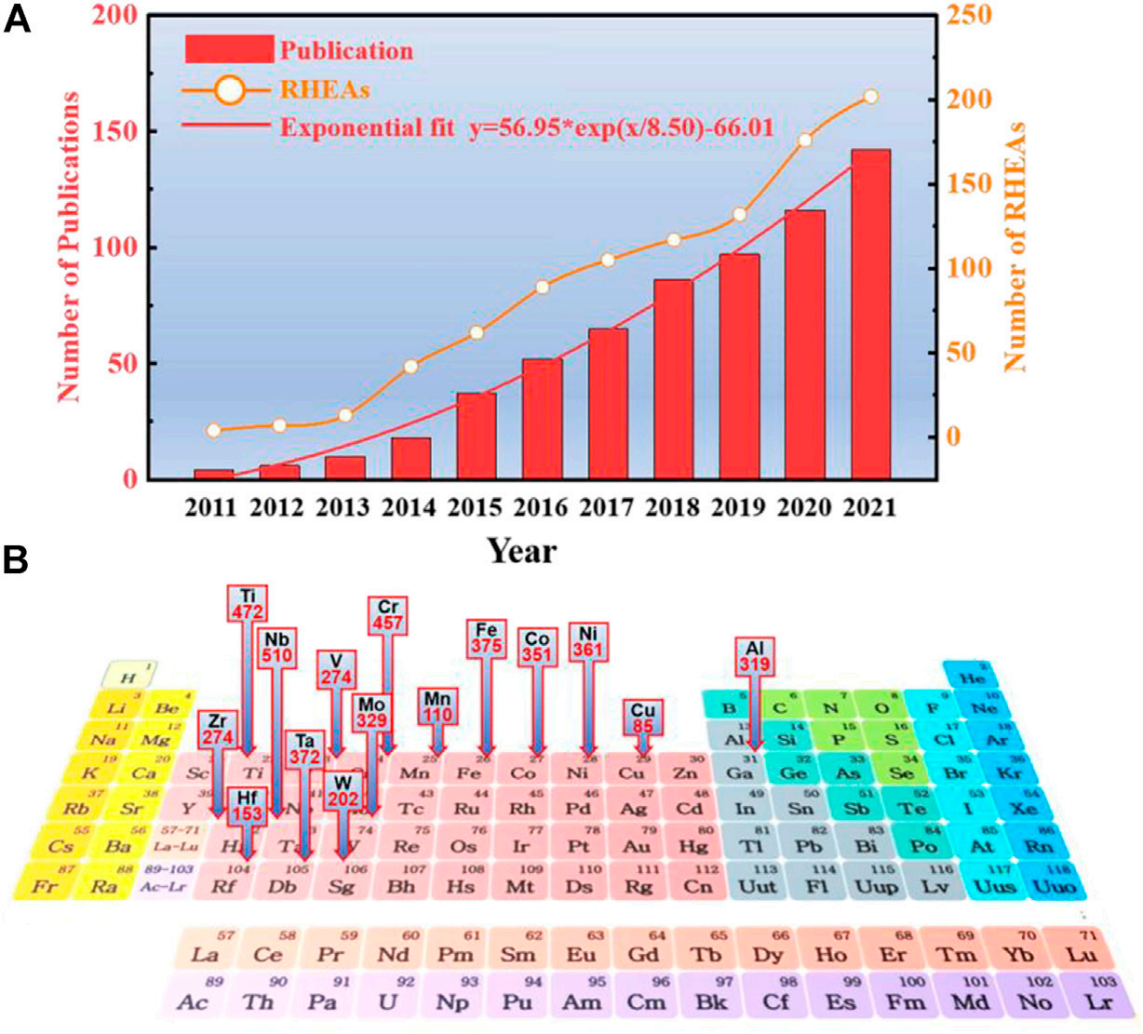
**(A)**. The number of RHEA publications from 2011 to 2021 and the frequency of RHEA occurrences involved. The number of RHEA articles can be described by exponential growth y=56.95∗exp(x/8.50)−66.01 . **(B)**. The frequency of occurrence and the distribution of their positions on the periodic table of elements in the 953 RHEAs counted.

**FIGURE 4:**
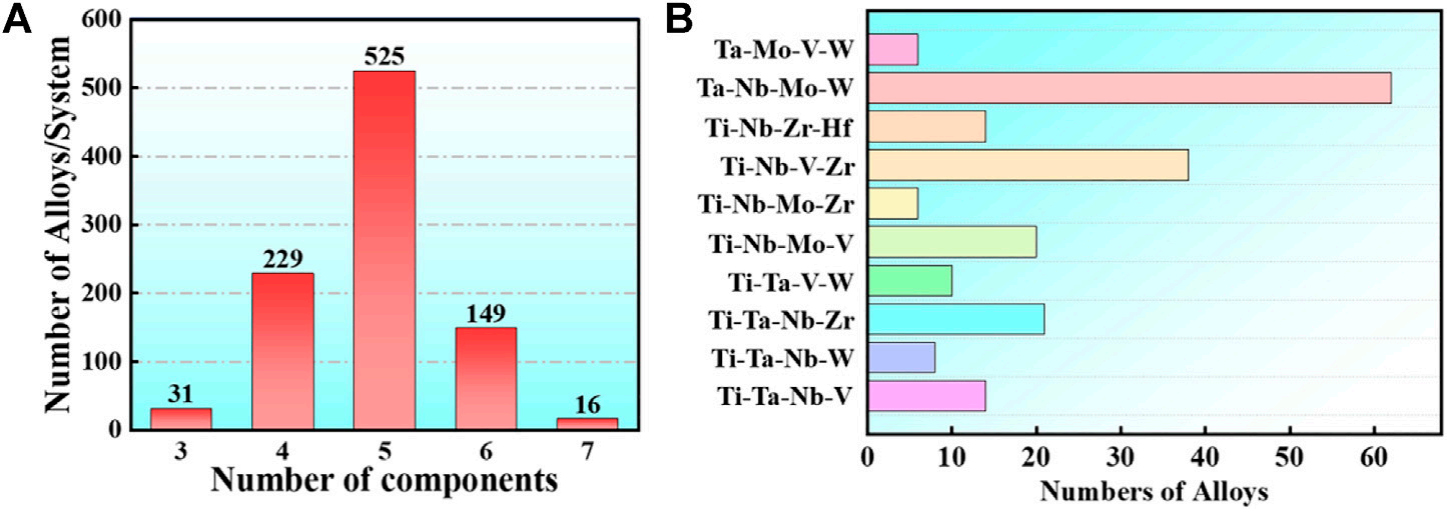
In the statistical publications, the frequencies of RHEAs of 3,4,5,6,7 group elements, respectively **(A)**, while the frequencies of RHEAs based on the most common 4 group element systems of Ta-Nb-Mo-W, Ti-Nb-V-Zr, Ti-Nb-Mo-V, and Ti-Ta-Nb-V are in **(B)**.

Bio-HEA alloys were developed based on the research of refractory high-entropy alloys (RHEAs). In 2010, Senkov et al. (Senkov et al., 2010) synthesized near-equivalent atoms WTaMoNb and WTaMoNbV alloys with single-phase body-centered cubic (BCC) lattice using the vacuum arc melting technique. Since then, many other HEAs based on refractory elements (Ti, Zr, Hf, V, Nb, Ta, Cr, Mo, and W) have been the focus of experimental studies (Gu P. et al., 2022; Chen S. H. et al., 2022; Huang et al., 2022; Peng et al., 2022; Zhou et al., 2022). The original RHEA was designed based on five refractory elements (Ta, Nb, Mo, W, and V). The broader elemental system of RHEAs includes Group IV (Ti, Zr, and Hf), Group V (V, Nb, and Ta), and Group VI (Cr, Mo, and W). There are also non-refractory metals such as Al, Si, Co, Cu, or Ni; the number of studies on RHEA is steadily increasing, as shown in Figure 3. Overall, the number of publications shows a roughly exponential relationship with year trends: y=56.95∗exp(x/8.50)−66.01 . By the end of December 2021, described 953 RHEAs alloys involved in 633 studies, among which 31 are 3-component, 229 are 4-component, 525 are 5-component, 149 are 6-component and 16 are 7-component (Figure 4). The most common elements in RHEAs are Nb(present in 510 alloys),Ti (472), Cr (457), Fe (375), Ta (372), Ni(361), Co(351), Mo (329), Al (319), V (274), Zr (274), W (202), Hf(153), Mn (110), Cu(85), and some other non-metallic elements Si, C and N are also included, providing more ideas for the composition design of HEA.

Bio-HEAs are based on the study of RHEAs, and the selected metal elements require no cytotoxicity or low cytotoxicity. The following eight alloy compositions are commonly used: Ti, Ta, Nb, Mo, V, W, Hf, and Zr. The refractory high-entropy alloys with four or more group elements are usually designed based on Ta-Nb-Mo-W, Ti-Nb-Zr-Hf, Ti-Nb-V-Zr, Ti-Nb-Mo-V, Ti-Ta-Nb-Zr, and Ti-Ta-Nb-V, as shown in Figure 4. In addition, alloys can contain Al or Si to reduce the alloy density to improve Bio-HEAs performance, and there are also studies to improve the wear performance of Bio-HEAs by adding Cr elements (Tong et al., 2020).

### Composition design theory

Bio-HEAs have excellent mechanical properties, and their strong solid solution strengthening mechanism plays an important role. The phase formation mode of HEAs was investigated to explore the composition design of different HEAs and many empirical parameters, such as atomic radius difference, mixing entropy, valence electron concentration, mixing enthalpy, etc. These parameters are usually constructed based on different perspectives such as thermodynamics, lattice distortion, and electronic behavior to explain the stability of the solid solution phase in HEAs. These empirical parameters can distinguish solid solution from intermetallic compound phases, discriminate the formation of phases such as FCC/BCC/HCP, and predict the single-phase and multi-phase structures of HEAs [8]. The researchers have summarized a large amount of data and proposed some semi-empirical criteria for the formation of simple solid solutions:

(1) The mixing entropy (ΔS_mix)_ is 12–17.5 J/(molK), and the calculation formula of ΔS_mix_ is (Zhou et al., 2022):

$$\Delta S_{mix}=-R\sum_{i=1}^{n}c_{i}lnc_{i}$$ (1)

where R is the gas constant, and c_i_ is the mole fraction of the *i*th element.

(2) The mixing enthalpy (ΔH_mix_) is −15–5 kJ/mol, and the calculation formula of ΔH_mix_ is (Zhou et al., 2022):

$$\Delta H_{mix}=-R\sum_{i=1}^{n}4H_{AB}c_{i}c_{j}\left(\text{i}\neq \text{j}\right)$$ (2)

where c_j_ denotes the mole fraction of the *j*th element; H_AB_ is the enthalpy of mixing between the A and B elements.

(3) The atomic size difference δ ≤ 6.5%, and the calculation formula of δ is (Zhou et al., 2022):

$$\delta =\sqrt{\sum_{i=1}^{n}c_{i}{\left(1-\frac{r_{i}}{\bar{r}}\right)}^{2}}$$ (3)

where r_i_ denotes the atomic radius of the *i*th component; is the molar average atomic radius.

In addition, the formulae for calculating the three relevant features of HEAs are added (Lilensten et al., 2018), including valence electron concentration (VEC), electronegativity difference (Δχ), and melting temperature (T_m_), and the calculation formula is as follows:

$$VEC=\sum_{i=1}^{n}c_{i}VEC_{i}$$ (4)

$$\Delta \chi =\sqrt{\sum_{i=1}^{n}c_{i}{\left(\chi_{i}-\bar{\chi }\right)}^{2}}$$ (5)

$$T_{m}=\sum_{i=1}^{n}c_{i}T_{mi}$$ (6)

Where c_i_ denotes the atomic concentrations for the *i*th element, VEC_i_ is the valence electron concentration of the *i*th element. T_mi_ is the melting point and Pauling electronegativity of the *i*th element. The structure and properties of Bio-HEA elements are listed in Table 1.

**TABLE 1:** Structure and properties of Bio-HEA elements.

Metals	T_m_(K)	VEC	r(Å)	Structure at RT	Structure at T_m_	χ	ρ(g/cm^3^)
Ti	1941	4	1.47	HCP	BCC	1.54	4.50
Zr	2,128	4	1.6	HCP	BCC	1.33	6.50
Hf	2,506	4	1.56	HCP	BCC	1.30	13.31
Nb	2,750	5	1.47	BCC	BCC	1.60	8.57
Ta	3,290	5	1.47	BCC	BCC	1.50	16.65
V	2,183	5	1.35	BCC	BCC	1.63	5.96
W	3,695	6	1.37	BCC	BCC	2.36	19.35
Mo	2,896	6	1.4	BCC	BCC	2.16	10.22

**FIGURE 5:**
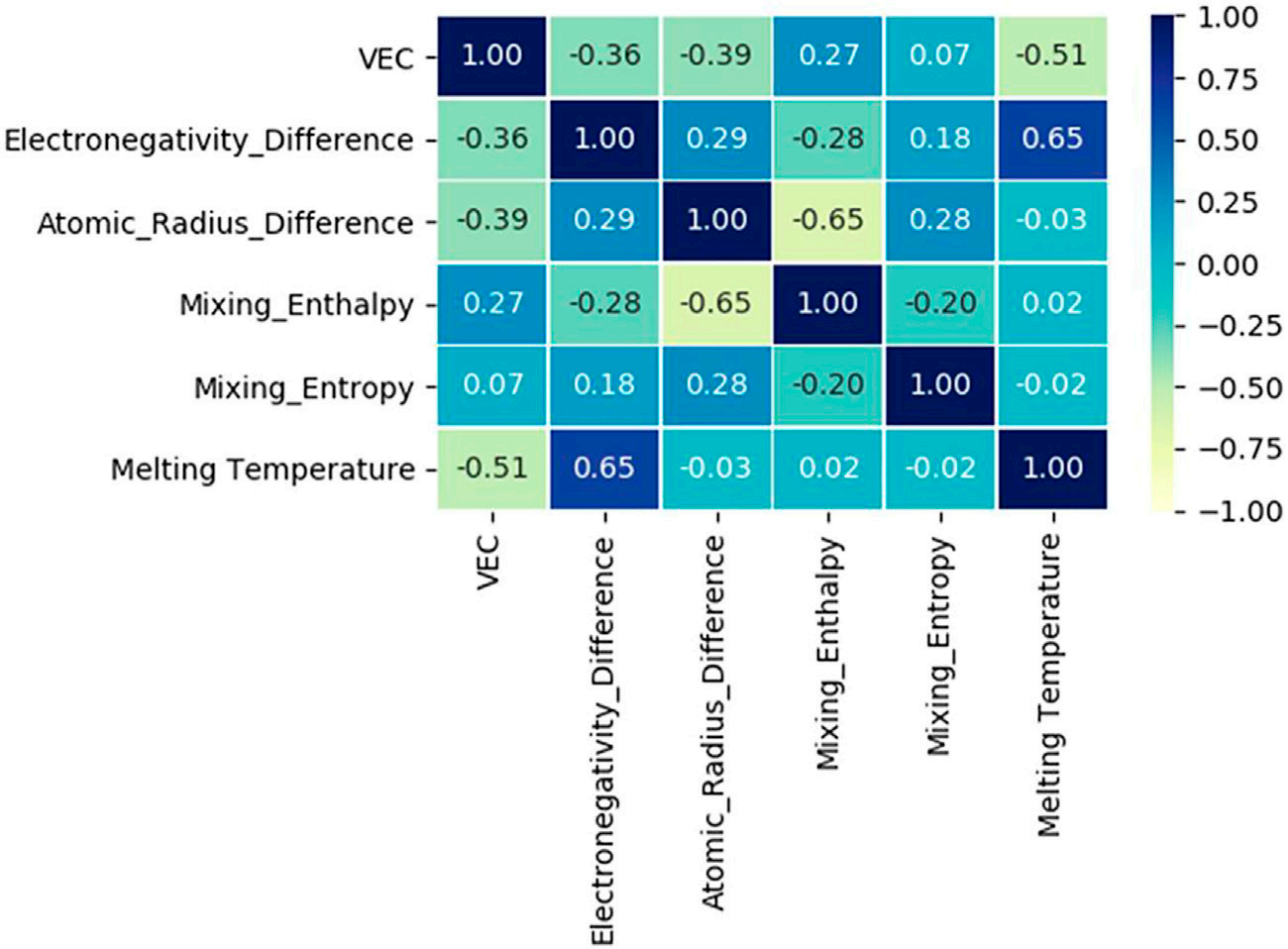
Heatmap of the correlation matrix between six relevant features, including VEC, electronegativity difference, mixing enthalpy, atomic size difference, mixing entropy, and melting temperature (Zhu et al., 2022).

Based on the six eigenvalues of the designed HEAs, Zhu et al. (Zhu et al., 2022) used an Artificial neural network (ANN) to count a dataset containing 529 HEAs. The Pearson correlation coefficients between the six eigenvalues are listed in Figure 5. The values in the matrix describe the correlation of the two eigenvalues, and the correlations quantitatively vary from 1 to −1, indicating a highly positive or negative relationship (After removing the autocorrelation value of 1, the matrix elements range from −0.65 to 0.65). For example, the atomic size difference is negatively correlated with the mixing enthalpy, which means that as the mixing enthalpy decreases, the atomic size difference appears larger. The electronegativity difference is positively correlated with the melting temperature. Overall, the absence of a strong positive or negative correlation matrix between any two features implies that each of these six feature values has a unique influence on the final properties of the alloy, which needs to be taken into account when designing new group element alloys.

### Solid-solution phase formation rules

**FIGURE 6:**
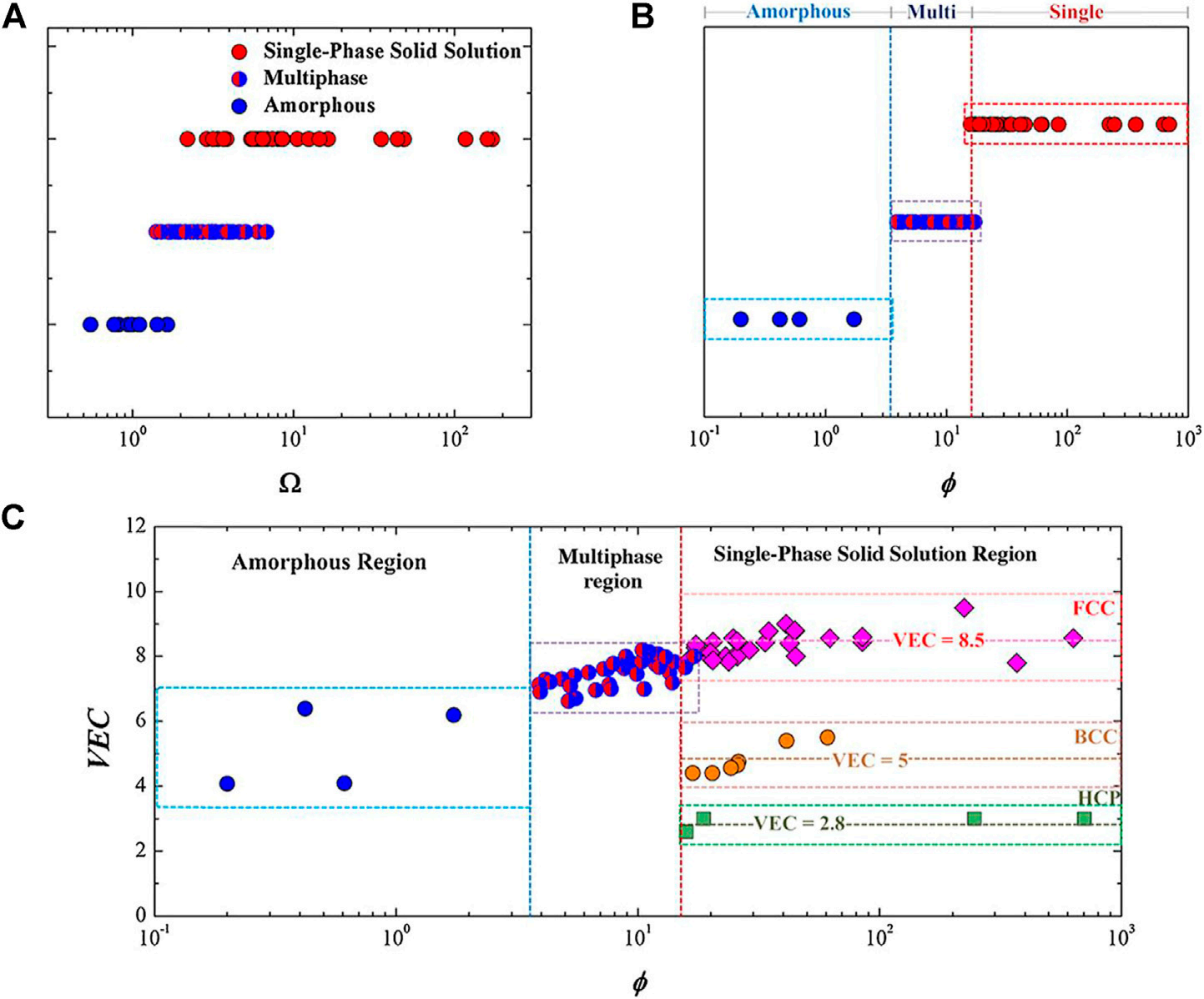
The plot of **(A)** the Ω values versus phases and **(B)** the ϕ values versus phases for the HEAs. **(C)** The plot of the VEC versus ϕ for different HEAs. The FCC solid solution mainly forms around a VEC of 8.5, BCC around a VEC of 5, and HCP around a VEC of 2.8, each within a narrow band (Ye et al., 2016)

The solid solution-phase structure of Bio-HEAs is a hot topic of research. For conventional binary solid solution materials, the classical Hume-Rothery criterion predicts the phase composition of the alloy from the atomic size, electronic structure, and other elemental properties (Ye et al., 2016). The alloys should have a high mixing entropy, low atomic size difference, and mixing enthalpies with small absolute values to form solid solutions (Zhang et al., 2008). In addition to the Hume-Rothery criterion, materials workers have proposed many simple but practical parameters to determine the phase composition tendency of high entropy alloys according to the properties of the alloy’s atoms. From Figure 6, it can be seen that Ω and ϕ affect the phase of HEAs, respectively, and a single eigenvalue cannot predict the trend of solid solution phase formation of HEAs. Still, it should be combined with the interaction of multiple eigenvalues. For example, several criteria have been proposed by experts and scholars to predict the formation conditions of solid solutions effectively. Zhang et al. (Zhang et al., 2008) indicated that the favorable conditions for the formation of the solid solution phase are −20 ≤ ΔHmix ≤5 kJ/mol, δ ≤ 6.4, and 12 ≤ ΔSmix ≤17.5 J/(mol K). Guo et al. (Guo et al., 2013) demonstrated that the parameter ranges for forming the solid solution phase are −11.6 ≤ ΔHmix ≤3.2 kJ/mol and δ ≤ 6.6. Yang and Zhang (Yang and Zhang, 2012) noted that the favorable criteria for predicting the formation conditions of the solid solution phase are Ω ≥ 1.1 and δ ≤ 6.6. The possibility of forming a solid solution phase can be notably enhanced during the design process of HEAs, reflecting on the criteria as mentioned above.

**FIGURE 7:**
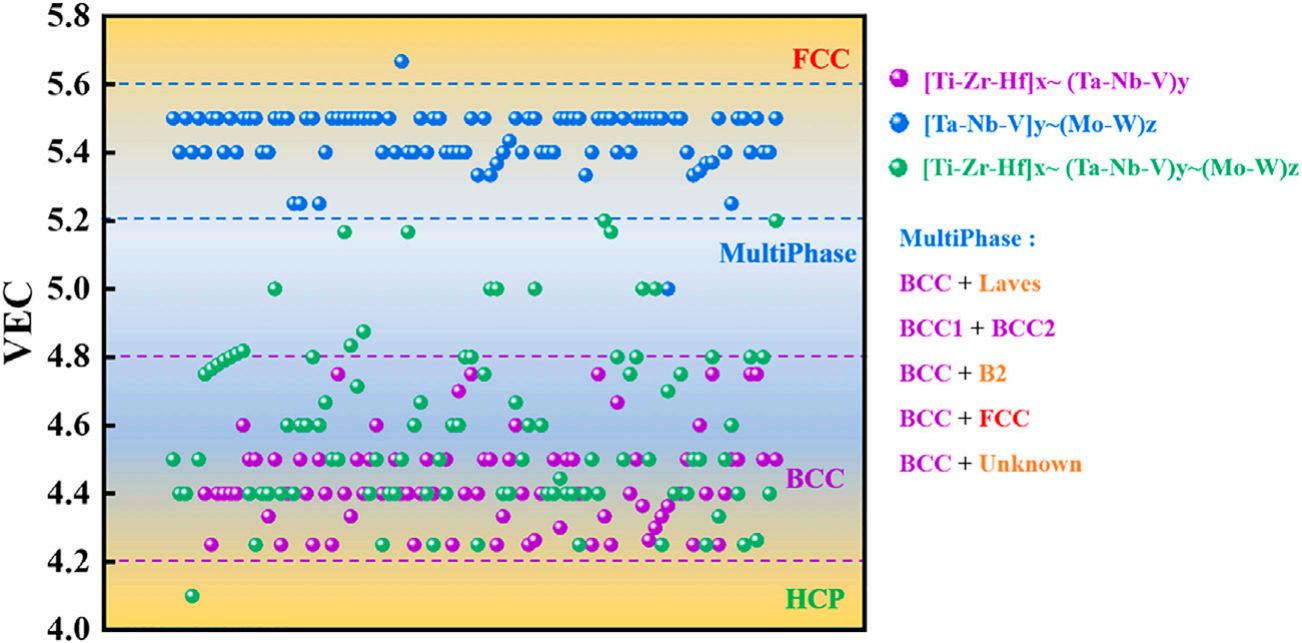
Statistical correspondence between VEC and phase composition in Bio-HEAs, Bio-HEAs are classified by [Ti-Zr-Hf]_x_∼ (Ta-Nb-V)_y_ [Ti-Zr-Hf]_x_∼ (Mo-W)_z_ and [Ti-Zr-Hf]_x_∼ (Ta-Nb-V)_y_∼ (Mo-W)_z_, where x,y,z ≠ 0.

For the whole alloy system of HEAs, the valence electron concentration (VEC) is the key parameter affecting the crystallization of the solid solution phase in the absence of strong atomic size effects, and a generalization work on the impact of VEC on Bio-HEAs in the literature is presented. To reduce the difficulty of computational work caused by multicomponent alloys, we use the cluster formula approach A cluster formula approach to simplify the calculation based on chemical short-range orders (CSROs). We can consider the solute atoms with similar properties in a multicomponent alloy as a cluster and the remaining solvent atoms with similar properties as a class of gum atoms, and most of the HEAs can be expressed as [cluster](gum atom)_x_ (Yang W. et al., 2022). In Bio-HEAs, the BCC solid solution structure is predominant, guided by Friedel oscillation theory, the gum atomic number is ideally calculated as x = 1–5, with a stable electronic structure. In the Bio-HEAs system, Ti, Zr, and Hf could be regarded as an averaged virtual element, M, since they are in the same group in the periodic table of elements, the value of VEC is four, and the ΔH_Ti-Zr_, ΔH_Ti-Hf_, and ΔH_Hf-Zr_ are zero. Ta, Nb, and V can also be considered an average virtual element, A, while Mo and V can be regarded as another average virtual element, B. We use M_x_A_y_B_z_ to generalize all [Ti-Zr-Hf]_x_∼ (Ta-Nb-V)_y_∼(Mo-W)_z_, and based on existing studies, we make a classification including [Ti-Zr-Hf]_x_∼ (Ta-Nb-V)_y_ [Ti-Zr-Hf]_x_∼ (Mo-W)_z_ and [Ta-Nb-V]_y_ ∼(Mo-W)_z_, and [Ti-Zr-Hf]_x_∼ (Ta-Nb-V)_y_∼(Mo-W)_z_, where x,y,z≠0. As can be seen from Figure 7, the VECs of the discussed Bio-HEAs range from 4.0 to 5.8, among which [Ti-Zr-Hf]_x_∼ (Ta-Nb-V)_y_ fluctuate between 4.2 and 4.8 due to the generally low VECs of their constituent elements, and are primarily single BCC solid solution phases. The VEC of Bio-HEAs [Ta-Nb-V]_y_ ∼(Mo-W)_z_ in the high VEC element group is concentrated in the range of 5.2–5.6, mainly in the BCC phase and multiphase, where the multiphase components include Laves phase, BCC2 phase, B2 phase, FCC phase and other precipitated phases (Wang et al., 2020; Mao et al., 2022). The interesting point is that alloys of the system [Ti-Zr-Hf]_x_∼ (Mo-W)_z_ hardly appear in the surveyed literature, implying that for Bio-HEAs, the V-subgroup elements Ta, Nb, and V with BCC lattice and high mutual solubility are indispensable.

The relationship between VEC and the solid solution phase has been studied thoroughly throughout the high entropy alloy system. The VEC of the alloy plays a crucial role in determining the crystallinity of the solid solution phase, especially for single-phase solid solutions. As mentioned earlier, the predominance of the BCC phase in the reported Bio-HEAs is quite reasonable, as the alloy is based on the V-subgroup elements and the IV-subgroup elements (Ti, Zr, and Hf), which are also BCC-phase structures at high temperatures. However, they undergo isotropic transformations and are HCP structures at RT, like Ti and Zr, as shown in Table 1. The phases included in the high VEC Bio-HEAs vary depending on the elemental composition and ratio and the effects of the process. Since the elements used are BCC, the generated solid solution phases are still based on the BCC phase, and the synergistic precipitation phases contain other solid solution phases such as Laves phase, BCC2 phase, B2, and FCC phase (Wei et al., 2022b).

The molybdenum equivalent (Mo_eq_) has been widely used to measure the β-phase stability in the multicomponent Ti-based alloy quantitatively. In addition, it has been shown that the Mo_eq_ parameter is also reliable for predicting the phase stability of solid solution in HEA, especially for the BCC/HCP phase (Wang et al., 2015); Ishida (Ishida, 2017) suggested a new Mo_eq_ based on the thermodynamic database of the Ti alloys, as shown in Eq. 7

$$\begin{array}{l} Moeq=\left[Mo\right]+0.26\left[Au\right]+0.43\left[Bi\right]+12.62\left[Be\right]+2.93\left[Co\right]+1.65\left[Cr\right]+\\ 0.85\left[Cu\right]+4.17\left[Fe\right]+0.05\left[Hf\right]+0.17\left[Mg\right]+3.28\left[Mn\right]+0.64\left[Nb\right]+\\ 1.75\left[Ni\right]+0.23\left[Os\right]+0.71\left[Pd\right]+0.64\left[Pt\right]+0.29\left[Pu\right]+1.72\left[Re\right]+2.89\left[Rh\right]+\\ 1.67\left[Ru\right]+0.97\left[Si\right]+0.23\left[Ta\right]+0.32\left[U\right]+0.80\left[V\right]+0.56\left[W\right]+1.13\left[Y\right]+\\ 0.16\left[Zr\right] \end{array}$$ (7)

**FIGURE 8:**
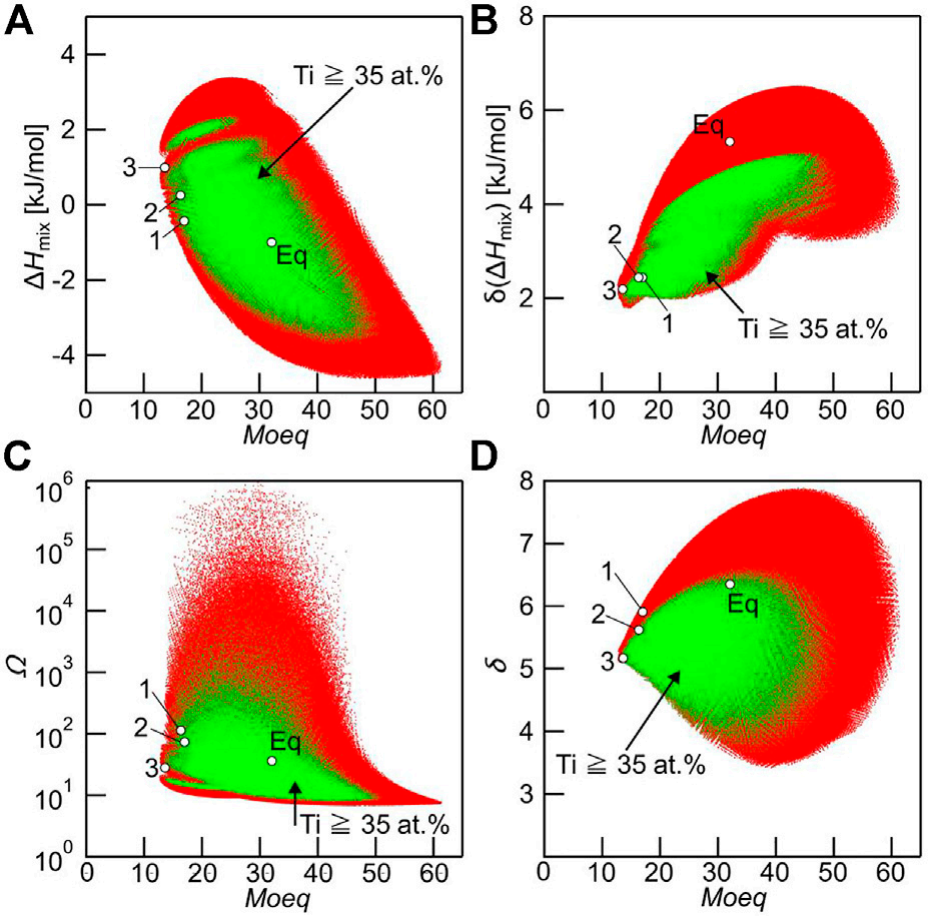
Relationship between Mo_eq_ and various empirical alloy parameters used to predict solid solution formation, including ΔHmix, δ (ΔHmix), Ω, and δ parameters in Ti-Zr-Hf-Nb-Ta-Mo alloys with ΔSmix ≥1.5 R (red dots). The green dot indicates the alloys whose Ti concentration was at and above 35 at% and considered the Ti-rich alloys. Hollow black circles (○) indicate the Ti–Zr–Hf–Nb–Ta–Mo alloy investigated in the present study. **(A)** Mo_eq_ vs ΔH_mix_, **(B)** Mo_eq_ vs δ(ΔH_mix_), **(C)** Mo_eq_ vs Ω, and **(D)** Mo_eq_ vs δ parameters (Iijima et al., 2021).

Where [M] is the weight percent concentration of element M. The inclusion of Ti, Zr, Hf, Nb, Ta, V, Mo, and W, the basic elements of Bio-HEAs, implies that the Mo_eq_ index is a guide to determining the desired stability of the solid solution phase in Bio-HEAs. Yang et al. (Yang W. et al., 2022) used Mo_eq_ to predict the phase stability of BCC/HCP solid solution in the Ti-Zr-Hf-Nb-Ta HEAs system. According to Yang’s calculations, the values of Mo_eq_ for Ti-Zr-Hf-Nb-Ta HEAs were in the range of 13.5–21.1 wt%, representing a high trend of BCC solid solution phase formation, which is consistent with the actual results. Iijima et al. (Iijima et al., 2021) used the Mo_eq_ parameter to predict various characteristic quantities of solid solution formation, including ΔH_mix_, Ω, and δ parameters, as shown in Figure 8. The calculations included 1.5×10^7^ Ti-Zr-Hf-Nb-Ta-Mo Bio-HEAs, and it was found that there was no clear relationship between the Mo_eq_ parameter and other eigenvalues. The Mo_eq_ parameter can be considered independent of other empirical alloy parameters, which can directly be predicted for the Bio-HEAs solid solution phase.

### Simulation research

Bio-HEA has a “cocktail” effect and is characterized by a multi-component synergistic effect. The design and development of new Bio-HEAs biomaterials require continuous changes in elemental composition and content (Li J. et al., 2021; Ostovari Moghaddam et al., 2021). However, Bio-HEAs have too many permutations of elemental composition and content ratios, and the elements used contain and are not only Ti, Zr, Hf, Nb, Ta, Mo, V, and W, which are expensive. Therefore, direct experimental verification of Bio-HEA alloys’ design is laborious and expensive. DFT has become one of the main methods for exploring material properties in theoretical solid-state physics. Over time, DFT calculations have entered the Bio-HEA field, and the number of corresponding studies has gradually increased (Yang F. et al., 2022; Biermair et al., 2022). Simulations based on the first principles of DFT are an essential method for designing Bio-HEA compositions. They are widely used to predict material properties such as energy band density, electronic structure, and charge density from atomic and electrical scales (Yu et al., 2019; Zhao, 2020; Wu et al., 2022).

**FIGURE 9:**
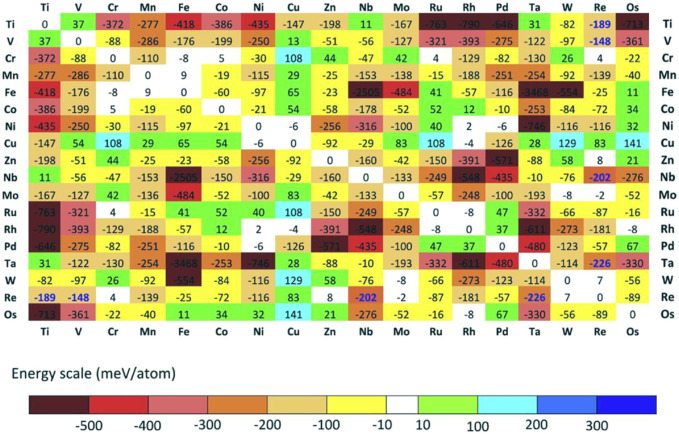
The DFT calculates the formation energy of the lowest energy structures in various binary relative to their phase separation into pure elements. If the numbers are in bold blue, the energies are evaluated relative to the respective solid solution (Troparevsky et al., 2015)

Troparevsky et al. (Troparevsky et al., 2015) evaluated the energy of formation of binary compounds by DFT calculations and therefore did not require experimentally or empirical inputs as shown in Figure 9. The model correctly ranks the combinations of elements of known single-phase HEAs based on statistics from numerous studies and eliminates combinations that are not single-phase. The constructed elemental matrix can predict all currently known single-phase HEAs. In addition, this method can predict feasible five-, six-, and seven-component alloys, thus guiding exploring new HEAs.

**FIGURE 10:**
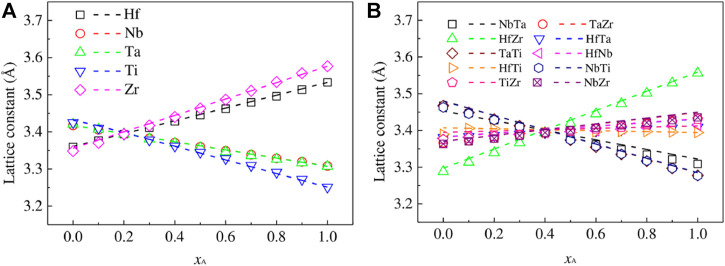
The final members of the lattice constants of the 1–4 **(A)** and 2–3 **(B)** pseudo-binary A_x_B_1- x_ alloys as a function of the molar fraction. In subfigure **(A)**, A represents one of the Hf-Nb-Ta-Ti-Zr elements, and B represents the other four elements. In subfigure **(B)**, A represents two elements of Hf-Nb-Ta-Ti-Zr, while B represents the other three. The lattice constants that conform to Vegard’s law are shown as dashed lines (Chen S. M. et al., 2022)

As the most important influencing factor of Bio-HEA biocompatibility, the selection of elements plays a significant role in the simulation design. Bai et al. (Bai et al., 2021) predicted the impact of Ti elements on the mechanical properties of iso-atomic NbMoTaW HEA from the first-principles calculation based on DFT. The phase structure, density, lattice constant, elastic, and electrical properties of NbMoTaW HEA by Ti elements were calculated. The energy band, total and partial densities of states, and charge density were calculated to investigate the strength and ductility enhancement mechanism of NbMoTaW alloy using Ti alloying. Tong et al. (Tong et al., 2020) used DFT calculations to simulate the effects of Ti, Zr, Cr, V, Hf, and Re additions on the properties of NbMoTaW HEAs and further analyzed the role played by various strengthening mechanisms. Chen et al. (Chen S. M. et al., 2022) used the first-principles approach combined with a thermodynamic model to study phase decomposition in alloys by considering HEA as various pseudo-binary systems, as shown in Figure 10, which predicts that phase decomposition in Hf-Nb-Ti-Zr alloys with a BCC structure occurs at temperatures below the critical temperature of 1298 K. The HEA decomposes most favorably into NbTa-rich and HfZr-rich BCC phases, while the BCC-rich HfZr phase is transferred to the hexagonal compact stacking structure (HCP) phase at low temperatures. (Chen X. et al., 2022; Chen Z. W. et al., 2022). In addition, the effects of solid solution and precipitation strengthening mechanisms on the strength of HEA were calculated based on the predicted phase decomposition results, combined with experimental data.

In the simulation studies of HEAs, apart from DFT simulations, molecular dynamics also occupy a relatively large proportion, and molecular dynamics-based simulations are more reliable in analyzing the microscopic deformation mechanism of HEAs. Progress has been made in using molecular dynamics simulations to explain the deformation mechanisms and mechanical properties of 3D transition group HEAs (Liu et al., 2020). This approach has significant implications for predicting the performance of HEAs, especially for assessing complex service conditions of Bio-HEAs. Simulation methods still have great research potential in the composition design of Bio-HEAs, and more research is needed to discover the intrinsic connections.

### Machine learning

With the increasing number of high-entropy components, the composition design becomes more and more complex, and the traditional empirical trial-and-error method, first-principles calculations, and molecular dynamics simulations introduced earlier gradually fail to meet the needs of the growing performance-oriented high-entropy alloy composition. Build machine learning models to predict the performance of various target alloys for fast and cost-effectively solved material performance evaluation (Kumar et al., 2021). Using a multi-objective genetic algorithm to find the Pareto Frontier of multi-objective performance in the prediction results can solve the problem of searching for elements of material composition (Bao et al., 2022). Combined with adaptive iterative methods for model uncertainty-based material screening, optimized material compositions or processes can be identified, guiding materials research (Chen H. et al., 2022; Thebelt et al., 2022). In the context of multi-objective performance requirements, the application of machine learning to the field of high-entropy alloys is particularly critical, such as the co-optimization of strength and corrosion resistance of high-strength and corrosion-resistant high-entropy alloys, the co-optimization of density and strong toughness properties of lightweight high-entropy alloys, the co-optimization of high-temperature strength and oxidation resistance properties needed for refractory high-entropy alloys, and the co-optimization of biocompatibility, mechanical properties matching those of living organisms, and excellent corrosion resistance required for biological high-entropy alloys.

**FIGURE 11:**
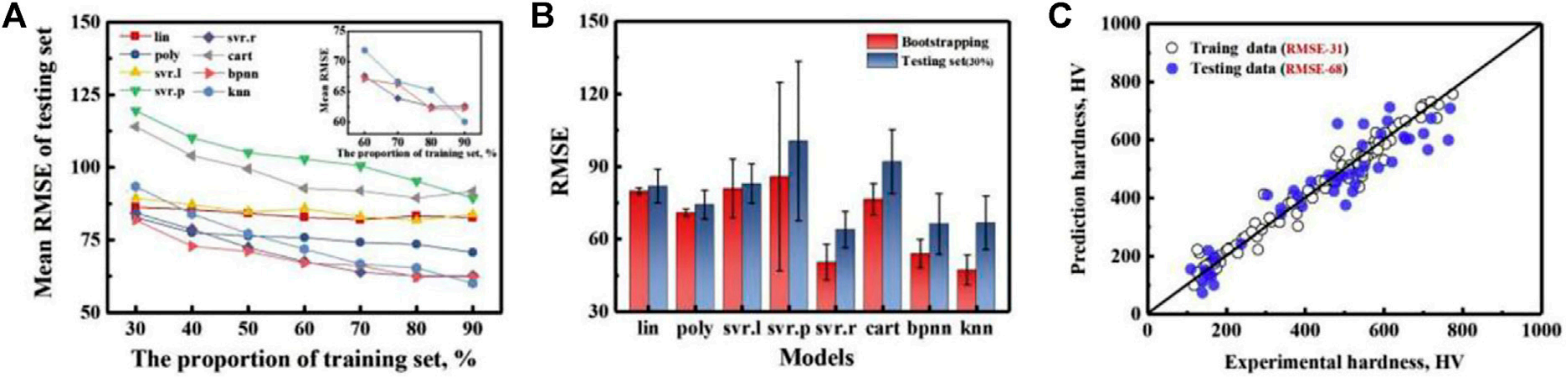
Test error evaluation of three different models, **(A)** a holdout method and **(B)** bootstrapping methods, indicating sur. r outperforms the bpnn and knn model. **(C)** The predicted values as a function of the measured values of the svr. r model for training data (randomly chosen 70%) and testing data (the rest 30%) (Wen et al., 2019)

Machine learning is applied to the strength and hardness properties of HEAs with data and set samples, either derived from experimental studies or obtained with the help of computational simulations (Bakr et al., 2022; Chen Z. W. et al., 2022; Hou et al., 2022). The research mainly focuses on machine learning to accurately predict the strength and hardness properties of new alloys and to guide the design of alloy compositions based on the property prediction results. Klimenko (Klimenko et al., 2021) and Bhandari (Bhandari et al., 2021) et al. developed support vector machine and random forest machine learning models for accurate prediction of yielding of HEAs with an accuracy of more than 95% based on a sample of high-entropy alloy data with characteristic parameters such as composition, modulus, density, mixing entropy, and atomic radius difference of HEAs as inputs. Several well-known machine learning models, including a radial basis function kernel (svr. r), a regression tree model (cart), a back propagation neural network model (bpnn), and a k-nearest neighbor model (knn) to produce a non-convex input/output fitness function to estimate the hardness (Wen et al., 2019). As shown in Figure 11, 42 newly synthesized HEAs were designed based on machine learning and experimental feedback results, with 35 of them having alloy hardness values higher than the best values in the training dataset.

In addition to the prediction of properties such as strength and hardness of HEAs, machine learning has also done a lot of work on elastic properties, which are the most critical concern for medical high-entropy alloys, to achieve the design of Bio-HEAs compositions corresponding to modulus and strength requirements based on the model’s prediction of elastic properties. Chanda et al. (Chanda et al., 2021) trained a neural network model using 140 samples of high-entropy alloy data with seven characteristic quantities such as electronegativity difference, mixing enthalpy, and mixing entropy as inputs and predicted the alloy elastic modulus with an accuracy of 94%. Roy et al. (Roy et al., 2020) trained gradient boosted tree regression models based on 89 high-entropy alloy data samples with ten empirical parameters as input characteristic parameters to predict the elastic modulus for 26 equimolar high-entropy alloys with low, medium, and high entropy compositions of the MoTaTiWZr system, and the agreement with the experimental results was high. The correlation coefficients revealed that the enthalpy of mixing and alloy melting point is the most critical for the prediction of elastic modulus, which provides a theoretical database for the subsequent design of Bio-HEAs with specific Young’s modulus to solve the stress shielding problem.

**FIGURE 12:**
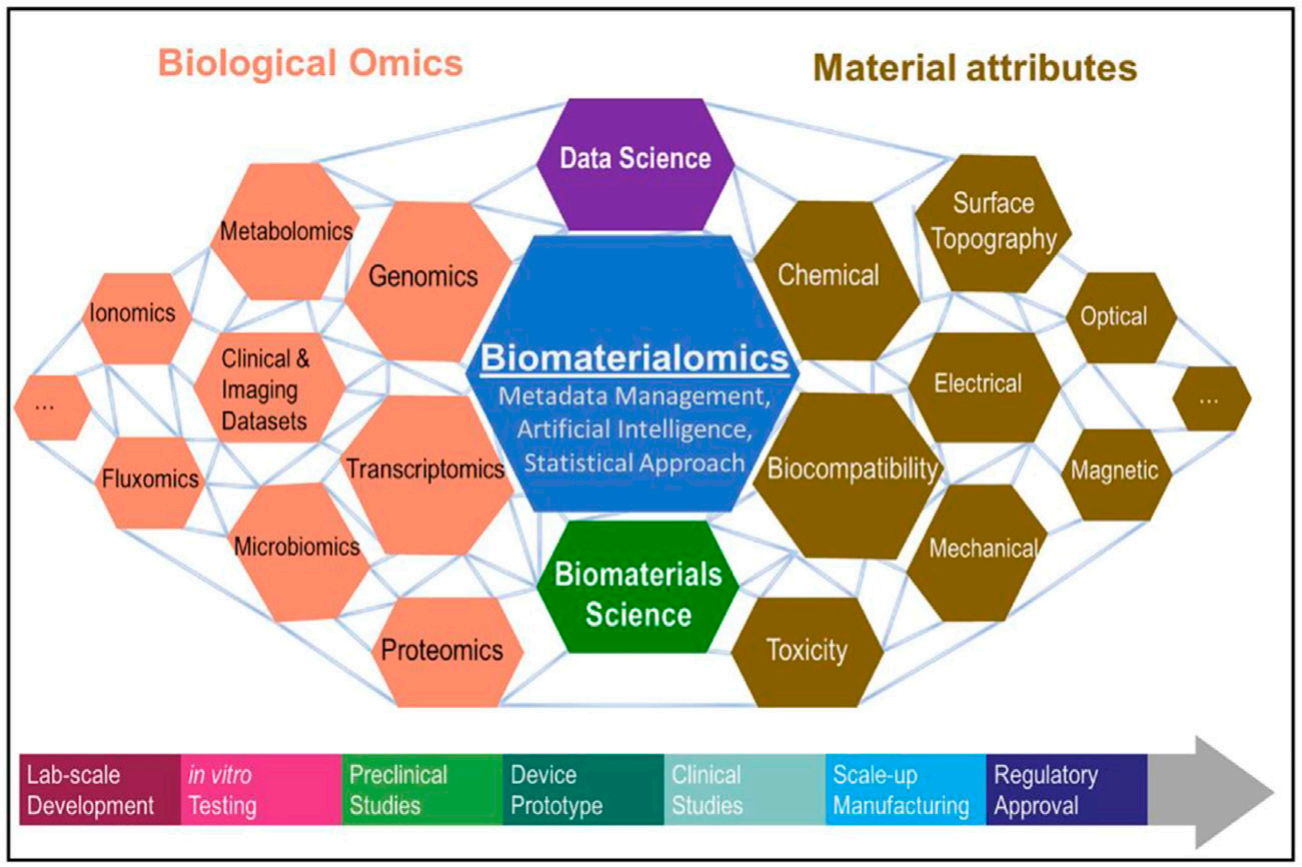
Introducing the concept of biomaterialomics, an interdisciplinary study unraveling the relationship between biological data and material properties (Basu et al., 2022).

As a new type of medical alloy, Bio-HEAs have the characteristics of biosafety, high strength, high corrosion resistance, high wear resistance, etc. It has recently become the most promising medical metal material for research (Liu et al., 2022b; 2022a). Biocompatibility is the focus of attention for Bio-HEAs in clinical applications, but the long development cycle of biomaterials (about 20 years) limits the speed of development of new materials. Rapid interaction between Biological Omics and Material attributes through machine learning will significantly shorten this process (Basu et al., 2022). It revealed the relationship between biological data and material properties through the concept of biomaterialomics, as shown in Figure 12. Therefore, material design measurement methods that integrate machine learning, search algorithms, and adaptive iteration are essential to guide the efficient design of Bio-HEAs with small data samples, large cost space, and multi-objective performance requirements.

## Biocompatibility

**FIGURE 13:**
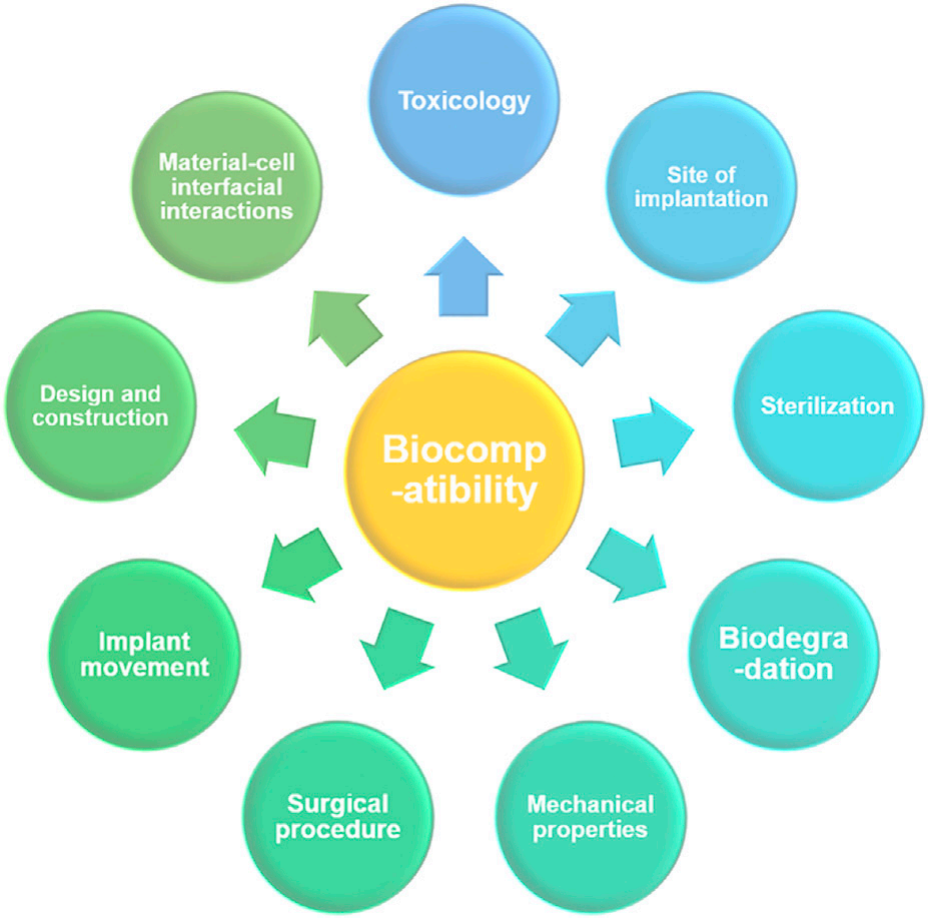
The primary factors are affecting the functional biocompatibility of a biomedical implant.

Due to the frequent occurrence of diseases, and other human injuries, the demand for implant materials has increased, and more attention has been paid to medical metal materials. In contrast, high-entropy alloys have become one of the most promising new medical materials in recent years due to their excellent performance and have been explored and researched by many scholars at home and abroad (Nagase et al., 2020). Bio-HEAs use Ti, Ta, Zr, Nb, and Hf elements with high biosafety and good biocompatibility. As a new bio-alloy material, Bio-HEAs require a series of biosafety assessments before they can be implanted in organisms, and the common factors affecting the biology of biomedical implants are shown in Figure 13. For Bio-HEA to be truly used in the clinical field as a biomedical implant, its biocompatibility needs to be experimentally proven by the mechanical, physical, and degradation properties, by sterilization (freeing the implant surface from all types of microorganisms), by toxicological aspects (examination and treatment of any toxins or toxic substances), by surgery, implant site and load-bearing capacity (Davis et al., 2022). Based on the existing research work, this chapter summarizes the biocompatibility performance of Bio-HEAs in terms of their cytocompatibility and corrosion resistance in physiological solutions.

### Cytocompatibility

Most of the research on Bio-HEA biocompatibility currently focuses on evaluating the cytocompatibility of HEA alloys by the direct cell contact method (Wang J. C. et al., 2022; Wei et al., 2022a; Guo et al., 2022). The cytocompatibility of alloy materials includes cell adhesion ability and cytotoxicity (Ma N. et al., 2020); cell adhesion is essential to maintain the stability of tissue structure and is a regulator of cell motility and function, with significant effects on cell proliferation and differentiation (Yang et al., 2011). Cytotoxicity is an indicator to assess the biological activity of cells on the surface of the alloy, which is one of the critical indicators for the *in vitro* evaluation of Bio-HEAs. The commonly used cell lines are human aortic smooth muscle cell, osteoblast, human osteosarcoma cell, human epithelial fibroblast, L929 mouse fibroblast cells, and Mouse embryo osteoblast precursor cells。

**FIGURE 14:**
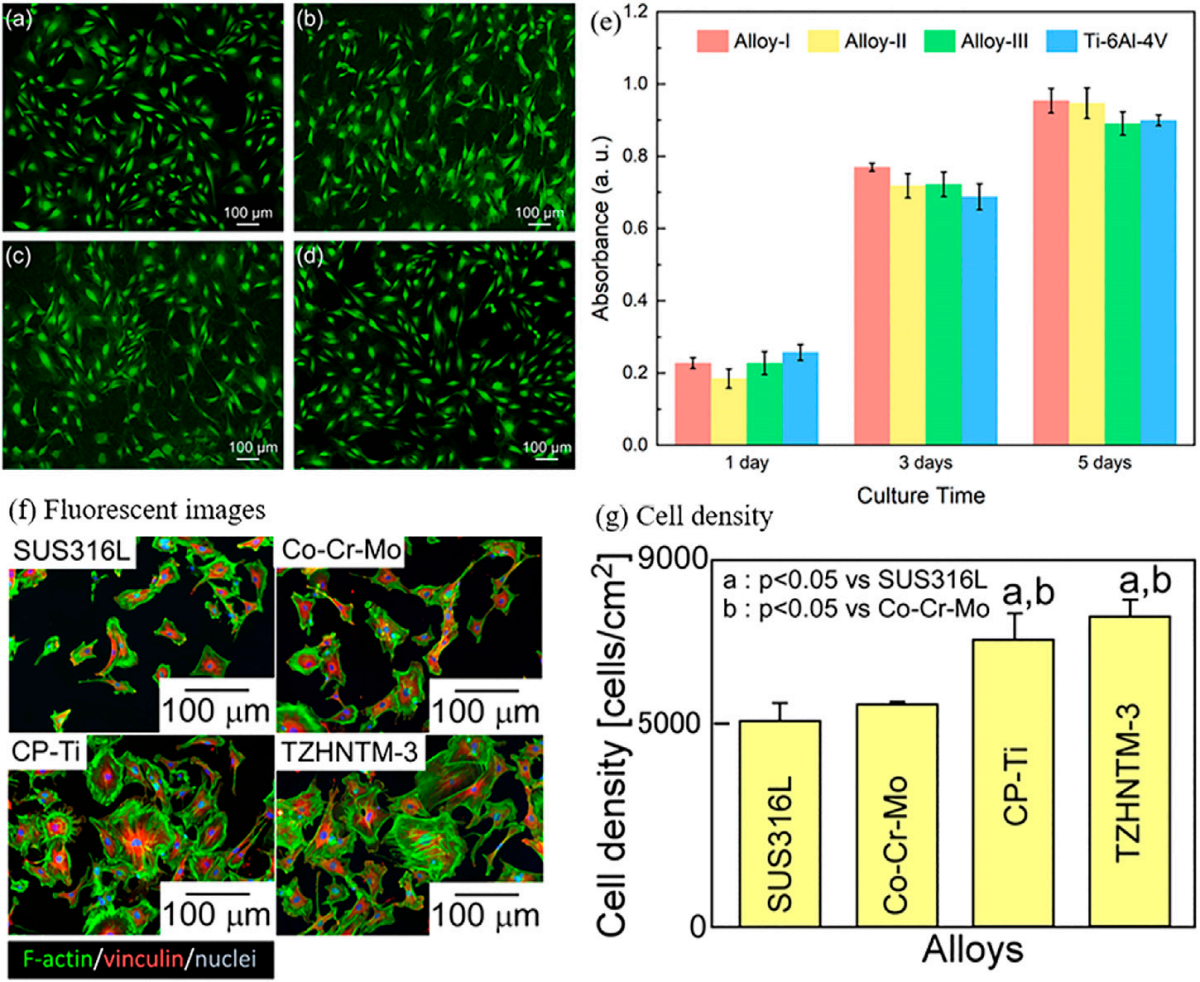
Live/dead staining of MC3T3-E1 cells on **(A)** Alloy-I, **(B)** Alloy-II, **(C)** Alloy-III, and **(D)** Ti64 after 72 h of incubation. **(E)** The proliferation of MC3T3-E1 cells on Alloy-I, Alloy-II, Alloy-III, and Ti64 after 1, 3, and 5 days of cell culture, respectively (Yang W. et al., 2022). Biocompatibility of the arc-melted isoatomic TiNbTaZrMo (TZHNTM-Eq) and non-isoatomic Ti_28.32_Zr_28.32_Hf_28.32_Nb_6.74_Ta_6.74_Mo_1.55_ (TZHNTM-3) and SUS316L stainless steel, ASTM F1537-08 of Co-Cr-Mo alloy and commercially pure titanium (CP-Ti) were used as references. **(F)** Quantitative analysis of the density of osteoblasts cultured on the fabricated specimens by staining images with Giemsa and **(G)** fluorescent images of osteoblast adhesion on the fabricated specimens (Iijima et al., 2021).

Currently, the cytotoxicity tests on Bio-HEAs are conducted by culturing cells *in vitro* and measuring their survival rate on the alloy surface to assess their biological properties. Todai et al. (Todai et al., 2017) cultured human osteoblasts on the surface of TiNbTaZrMo Bio-HEA, which exhibited superior biological activity to pure Ti. Yang et al. (Yang W. et al., 2022) used MC3T3-E1 cells on Ti_20_Zr_20_Hf_20_Nb_20_Ta_20_ (Alloy-I), Ti_25_Zr_25_Hf_25_Nb_12.5_Ta_12.5_ (Alloy-II), Ti_27.78_Zr_27.78_Hf_27.78_Nb_8.33_Ta_8.33_ (Alloy- III) and Ti6Al4V substrates were incubated to evaluate the biocompatibility of Ti-Zr-Hf-Nb-Ta HEA, as shown in Figure 14. The biological activities of HEA and Ti-6Al-4V were observed by fluorescence staining after 72 h of culture, as shown in Figure 14. It was found that a large number of MC3T3-E1 cells adhered to the surface of HEA and Ti-6Al-4V with a high survival rate of adherent cells, indicating that MC3T3-E1 cells had a high survival rate and good initial adhesion on this HEA. In addition, the survival numbers of cells in HEA and Ti-6Al-4V were counted on the first, second, and third days, respectively, and no statistically significant differences were found, indicating that TiZrHfNbTa HEA has the same level of biocompatibility as Ti-6Al-4V. Iijima et al. (Iijima et al., 2021) used mouse primary osteoblasts cultured on TiZrHfNbTaMo surface for 24 h. After 24 h in a humidified atmosphere of 5% CO2, the cells were fixed in methanol, stained with 5% Giemsa aqueous solution for staining, and observed under a light microscope, as shown in Figure 14. In addition, Bio-HEAs such as TiZrNbHfTa (Braic et al., 2012; Motallebzadeh et al., 2018) have good mechanical properties and excellent friction and wear resistance, showing close to or even better biocompatibility than conventional medical alloys such as Ti-6Al-4V.

### Corrosion resistance

Bio-HEAs have been shown to have excellent cytocompatibility. Still, the application of the material should also consider the working service environment, where all metals and alloys are subject to corrosion when in contact with body fluids because the body environment is very aggressive due to the presence of hydrogen ions and chloride ions, and proteins (Khodaei et al., 2020). Bio-HEAs, as metal implants, undergo various chemical reactions with body fluids, where the metal components of the alloy are oxidized to ionic form and dissolved oxygen is reduced to hydroxide ions, causing various forms of corrosion damage (Cui et al., 2022). For the study of the corrosion resistance of Bio-HEAs, the solutions commonly used to simulate the physiological environment include saline (0.9% NaCl solution), Hank’s solution (with a composition of 0.137 M of NaCl, 5.4 mM of KCl, 0.25 mM of Na_2_HPO_4_, 0.44 mM of KH_2_PO_4_, 1.3 mM of CaCl_2_, 1.0 mM of MgSO_4_, and 4.2 mM of NaHCO_3_, pH = 7.4), phosphate buffer saline (PBS) and fetal bovine serum (FBS).

Potentiodynamic polarization curve measurement is one of the most widely used measurements in electrochemical corrosion (Gu M. et al., 2022; Li et al., 2022), which can provide information on the corrosion rate and corrosion mechanism of bio-alloy materials under a simulated physiological environment and evaluate the bio-suitability of Bio-HEAs by chemical corrosion conditions. In the corrosion reaction, corrosion potential (E_corr_), corrosion current density (I_corr_), and AC impedance are essential parameters for analyzing the corrosion resistance of the material, and the relevant parameters of some Bio-HEAs are listed as shown in Table 2. For Bio-HEAs with Ti and Ta elements, their lower E_corr_ indicates that they will both readily form protective passivation layers (usually oxides) (Zheng et al., 2018). Most Bio-HEAs have no pitting reaction until 2V, which means promising applications in human environments around 0–0.2 V. No pitting and no localized corrosion due to the breakdown of the protective passivation films, dramatically increasing the lifetime of alloy implants (Hwang et al., 2019; Li T. et al., 2021). The corrosion current density indicates the corrosion rate of the material. The lower the I_corr_ value, the lower the corrosion rate of the material, and the low I_corr_ value of Bio-HEAs indicates that it is preferable in medical alloy implant applications (Zhu et al., 2021; Feng H. et al., 2022).

**TABLE 2:** Electrochemical parameters of the relevant Bio-HEAs in simulated human solutions.

Bio-HEA	Solution	E_corr_(V)	I_corr_ ( × 10^^−3^A/m^−2^)	Epit(V)	Refs
Ti_20_Zr_20_Hf_20_Nb_20_Ta_20_	Hank’s	−0.302 ± 0.014	0.8 ± 0.20		Yang et al. (2022b)
Ti_25_Zr_25_Hf_25_Nb_12.5_Ta_12.5_	Hank’s	−0.407 ± 0.021	1.19 ± 0.42		Yang et al. (2022b)
Ti_27.78_Zr_27.78_Hf_27.78_Nb_8.33_Ta_8.33_	Hank’s	−0.437 ± 0.062	1.22 ± 0.37	1.238	Yang et al. (2022b)
TC4	Hank’s	−0.347 ± 0.014	0.61 ± 0.14		Yang et al. (2022b)
Pure Ti film	Hank’s	−0.252	1.101	＞2	Lai et al. (2018)
Ta_57_Ti_17_Zr_15_Si_11_ film	Hank’s	−0.304	0.77	＞2	Lai et al. (2018)
Ta_75_Ti_10_Zr_8_Si_7_ film	Hank’s	−0.336	0.83	＞2	Lai et al. (2018)
Pure Ta film	Hank’s	−0.432	0.61	＞2	Lai et al. (2018)
TiZrNbTaMo	PBS	−0.607	0.3	2	Wang and Xu, (2017)
316L SS	PBS	−0.234		0.675	Wang and Xu, (2017)
CoCrMo	PBS	−0.320		0.435	Wang and Xu, (2017)
TiZrHfNbTa	Hank’s	−0.395	0.8		Yang et al. (2020)
TiZrHfNb	PBS	−0.39 ± 0.03	10.93 ± 2.77		Wang et al. (2022c)
TiZrHfNbFe_0.25_	PBS	−0.42 ± 0.05	9.33 ± 1.61		Wang et al. (2022c)
TiZrHfNbFe_0.5_	PBS	−0.30 ± 0.01	2.80 ± 0.77		Wang et al. (2022c)
TiZrHfNbFe_0.75_	PBS	−0.27 ± 0.01	1.66 ± 0.27	1.36 ± 0.02	Wang et al. (2022c)
TiZrHfNbFe	PBS	−0.33 ± 0.02	5.18 ± 1.81	1.22 ± 0.12	Wang et al. (2022c)
TiZrHfNbFe_1.5_	PBS	−0.49 ± 0.04	15.5 ± 0.25	1.16 ± 0.16	Wang et al. (2022c)
TiZrHfNbFe_2_	PBS	−0.51 ± 0.01	27.1 ± 2.21	0.82 ± 0.03	Wang et al. (2022c)
TiZrTaHfNb	PBS	−0.396	0.72		Motallebzadeh et al. (2019)
Ti_1.5_ZrTa_0.5_Hf_0.5_Nb_0.5_	PBS	−0.396	0.56		Motallebzadeh et al. (2019)

**FIGURE 15:**
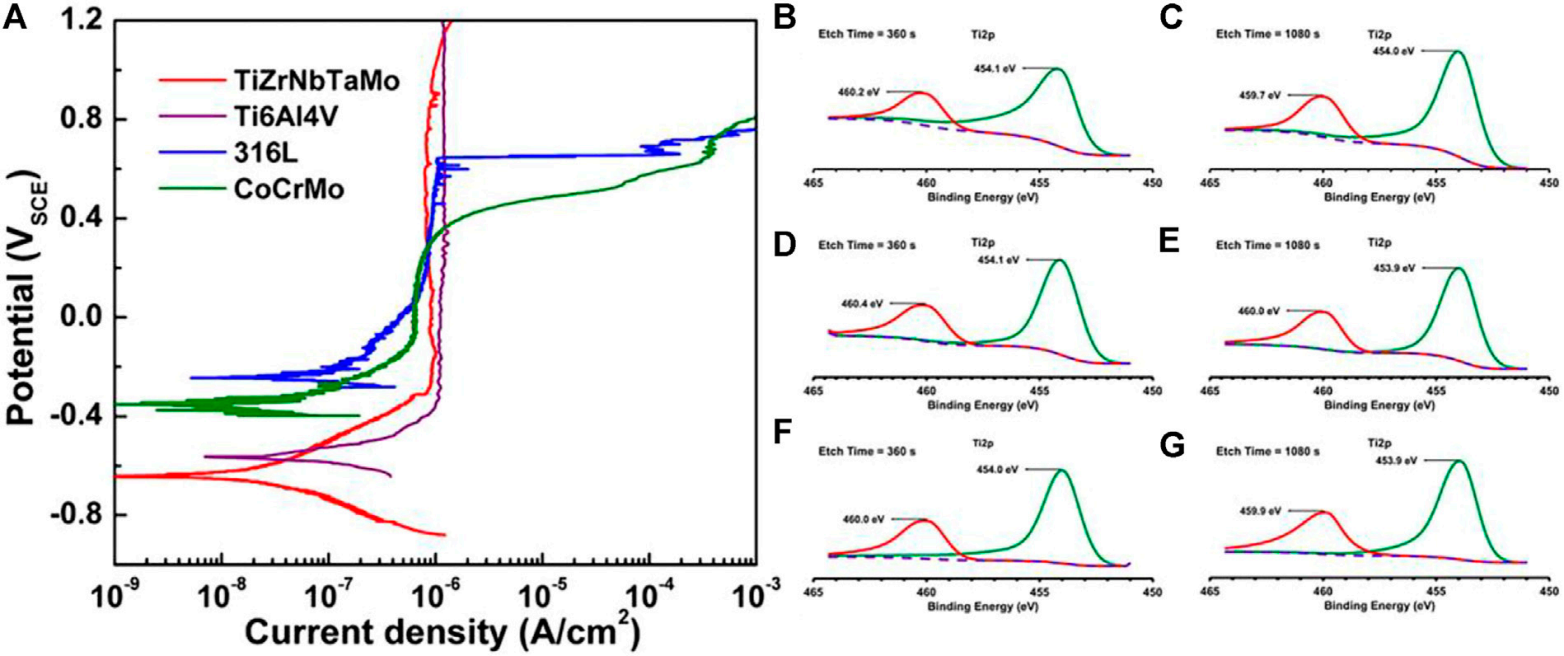
**(A)** Potentiodynamic polarization curves of arc-melted TiZrNbTaMo HEA, as well as Ti-6Al-4V, 316L, and CoCrMo alloys in PBS for comparison of biocompatibility (Wang and Xu, 2017). Ti2p peaks after etching times of 360 and 1,080 s immersed in FBS for 28 days: **(B)** and **(C)** for TiTaHfNb, **(D)** and **(E)** for TiTaHfNbZr, **(F)** and **(G)** for TiTaHfMoZr (Gurel et al., 2021).

The Ti-Zr-Ta-Hf-Nb system of Bio-HEAs has promising properties as a novel superior metallic biomaterial with an ideal combination of wear resistance, wettability, pitting, and resistance to general corrosion outperforming the conventional metallic biomaterials 316L, CoCrMo, and Ti-6Al-4V in these aspects (Cui et al., 2022). Wang et al. (Wang and Xu, 2017) investigated the electrochemical behavior of HEA in PBS by kinetic potential polarization test. They initially evaluated its corrosion resistance in a physiological environment and compared it with Ti6Al4V, 316L SS, and CoCrMo alloys, as shown in Figure 15. TiZrNbTaMo HEA exhibits an extensive passive plateau in the curve up to 1.2 VSCE without pitting or turning passivation. Such a response is similar to that of Ti-6Al-4V, except for a more positive E_corr_ and a slightly higher pass for Ti-6Al-4V. TiZrNbTaMo HEA exhibited excellent corrosion resistance comparable to Ti6Al4V alloy and significantly better-pitting resistance than 316 L SS and CoCrMo alloys in a physiological environment simulated by PBS media. TiZrNbTaMo HEA exhibited excellent corrosion resistance comparable to Ti6Al4V alloy and significantly better-pitting resistance than 316 L SS and CoCrMo alloys in a physiological environment simulated by PBS media. Gurel et al. (Gurel et al., 2021) studied the biocompatibility of three TiTaHf-based high-entropy alloys, namely TiTaHfNb, TiTaHfNbZr, and TiTaHfMoZr alloys, using FBS as a test environment to compare their corrosion resistance as bone implants by the level of ions released from HEA etched by FBS solution, TiTaHfNb HEA exhibited the highest corrosion resistance to FBS, as shown in Figure 15. In addition, after 28 days of immersion in FBS solution, the three HEA were found to produce hydroxyapatite on their surfaces when in contact with FBS, further demonstrating their great potential for use in orthopedic implants.

### Antibacterial property

In addition to applications in human implants, Bio-HEAs have great potential for medical devices. Conventional medical devices, despite their strict sterilization procedures, are still subject to bacterial infections, which greatly threaten the health of patients (Grischke et al., 2016; Zhang et al., 2021). Antibacterial metals and alloys are more suitable metal materials for medical devices, prepared by elemental alloying and processing processes, exhibiting strong inhibition of bacterial adhesion, growth, and proliferation. Currently, common antibacterial medical devices are antibacterial stainless steel, antibacterial magnesium alloy and antibacterial titanium alloy, and the elements that play an antibacterial role are mainly Cu, Ag and Zn (Chopra, 2007; Tie et al., 2013; Nan and Yang, 2016). Biological HEAs with antibacterial properties usually contain these antibacterial elements, and Chen et al. (Chen C. et al., 2022) designed CrFeNiCuSi HEA with antibacterial properties, which achieved 97.45% inhibition of *E. coli*. Similarly, the synergistic effect of copper ions and copper-rich phase greatly improved the antibacterial performance of Bio-HEAs, and CoFeCrCu HEA showed superior antibacterial performance with 99.97% inhibition of *E. coli* and 99.96% inhibition of *Staphylococcus aureus* after 24 h, much higher than conventional antibacterial alloy materials (Ren et al., 2022).

The antibacterial mechanism exhibited by Bio-HEAs containing Cu elements has been explored and verified (Sarell et al., 2010; Squitti et al., 2015; Zhang et al., 2019). On the one hand, the electrostatic force of Cu^2+^ can directly disrupt the adhesion of bacteria and rupture their cell walls, leading to cell rupture and death. On the other hand, in HEA, Cu and other elements (such as Fe) form a potential difference to form a miniature galvanic cell, releasing a large amount of Cu^2+^, which further exerts the bactericidal effect of Cu ions. In addition, according to Ren et al. (Ren et al., 2022), direct contact of bacteria with the copper-rich phase in Bio-HEAs produces an effective concentration of ROS (H_2_O_2_) during incubation, which induces oxidative stress, resulting in an antibacterial effect.

HEAs with antibacterial ability not only have strong and long-lasting bactericidal ability but also have good corrosion resistance and mechanical properties, indicating that Bio-HEAs also have great potential in the field of medical devices. Bio-HEAs containing Cu ions or Ag ions show stronger antibacterial ability, but the release of Cu^2+^ and Ag + may cause some damage to human body, which requires finding the right combination of elements to make Bio-HEAs have antibacterial ability and at the same time have basically no side effects on human body.

## Mechanical properties

In addition to the biological safety of the material, meeting specific medical functions is a fundamental requirement of Bio-HEA. The implant generally serves as a replacement for damaged bone or as extra support. For different disease sites, an alloy with mechanical properties appropriate to the tissue is required. The mechanical match between the material and the biological organism during the implantation and functionalization of the material also influences the service effect of the implant in the damaged area. Too low modulus of elasticity can reduce the medical therapeutic impact of the alloy, while the too high modulus of elasticity can lead to stress shielding problems (Uppal et al., 2022; Xing et al., 2022). Therefore, Bio-HEA should not be pursued for high-strength mechanical properties but should be consistent with human tissues, close to or complete the therapeutic effect before failure. The natural selection of Bio-HEAs for implants is due to the multiple biomaterials advantages, including high strength, low density (high specific strength), high corrosion resistance, complete inertness to the body environment, and enhanced biocompatibility, low modulus, and increased capacity to join with bone and other tissues. These properties fit with the specific properties of biomedical metal materials, which means there is a good potential for its application in the medical field.

### Strength and ductility

From an engineering perspective, the multi-component nature of HEAs predestines the strengthening mechanism of HEAs to be equally multidimensional. Current research on HEAs is characterized by numerous discoveries, intense discussions, and illuminating scientific research questions (Chen X. et al., 2022; Jin et al., 2022). Strength and ductility are widely studied as core mechanical properties of metallic materials. Complex alloying and thermo-mechanical processing affect the microstructure of HEAs, which has a dramatic effect on strength and ductility (Wu and Fan, 2020; He et al., 2021).

Several factors may influence the strength and ductility of Bio-HEA. Undoubtedly, the elemental composition is the most critical factor. Moreover, the strength and ductility may be changed by various factors such as microstructure, processing method, and post-heat treatment (Liu Z. et al., 2022; Jin et al., 2022). At the same time, these influencing factors interact with each other. For example, the microstructure manifests the combined effect of elemental composition and processing routes of Bio-HEAs. This composition-processing route-microstructure-property study idea, widely applied in conventional alloys, is also applicable in HEAs alloy systems. Still, the challenges are enormous, and the verification is more complex.

The elements composition of Bio-HEAs has the most critical effect on its strength and ductility. First, alloying elements determine the elastic behavior and atomic interactions of Bio-HEA, affecting the strength and ductility of the alloy at the atomic level. In addition, the ratio of elements can determine the phase composition and fraction of Bio-HEAs. In multi-component Bio-HEAs, the type and content of modulating elements can have a significant impact on the mechanical properties of the HEA, and common element additions include metallic and non-metallic elements (Tancret, 2021).

As the most common element in Bio-HEAs, Nb can provide an effective strengthening effect. The strengthening effect of Nb is due to its large atomic radius, which produces severe lattice distortion when added. It is widely used as a precipitation hardening element in the Bio-HEAs system (Li W. et al., 2021). Like Nb, Ti element with a larger atomic radius also strengthens solid solution in Bio-HEAs (Zhang et al., 2017). Still, the difference is that the strengthening effect of Ti on Bio-HEAs occurs mainly at low Ti content. Low Ti element concentration promotes solid solution formation, strengthened BCC phase, and hard Laves phase. In contrast, high Ti concentration leads to the precipitation of intermetallic compounds in HEAs to embrittle the alloy (Zhou et al., 2007). The strengthening mechanism of element Ta (Mohd Pauzi et al., 2016) for Al_0.5_FeCrNiMnCo (the primary phase is BCC + FCC) is mainly characterized by the precipitation strengthening effect and the presence of more Laves intermetallic phase precipitation. In addition, the strengthening effects of W (Waseem and Ryu, 2017), Zr (Yurchenko et al., 2017), and Cr (Stepanov et al., 2015) in Bio-HEAs were analyzed separately, and the compressive and tensile strengths increased to varying degrees with the addition of small amounts of the elements. Still, their strength benefits were compromised as the elements were alloyed. On the other hand, ductility tends to be the opposite of strength, and depending on the type of alloy, strength and ductility may vary roughly linear or nonlinearly with element concentration.

**FIGURE 16:**
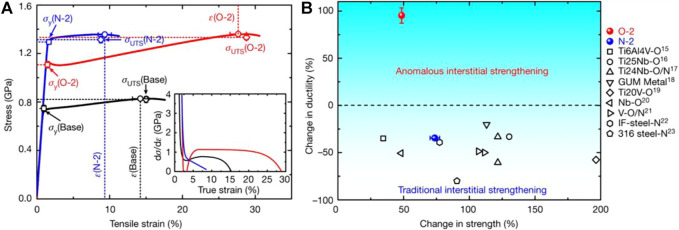
**(A)** Room-temperature tensile stress-strain curves for TiZrHfNb (TiZrHfNb)_98_O_2_, and (TiZrHfNb)_98_N_2_ HEA. The inset shows the corresponding strain-hardening response (dσ/dε) **(B)** Variation in strength and ductility of the HEA presented here, relative to several established high-performance alloys (Lei et al., 2018).

Small amounts of non-metallic elements, such as O, B, C, N, and Si, are usually added to Bio-HEAs to improve the overall performance of the alloy. According to the traditional experience, adding C elements to steel materials can greatly improve the mechanical properties of steel, and this method is also applicable to Bio-HEAs. The trace addition of C elements will form MC-type alloy carbides on the BCC matrix of Hf_0.5_Mo_0.5_NbTiZr, and the work-hardening ability and plastic strain of the alloy is enhanced (Gao et al., 2021). Chen et al. (Chen et al., 2018) investigated the effect of O element content on the microstructure and compressive properties of ZrTiHfNb_0.5_Ta_0.5_ Bio-HEAs. O atoms were present in the lattice of ZrTiHfNb_0.5_Ta_0.5_ Bio-HEAs and did not precipitate the MO alloy oxide phase. With the increase of the O element, the interstitial solid solution strengthening effect of the O atom increases, and the yield strength of the alloy gradually increases but the plasticity decreases. Further, Lei et al. (Lei et al., 2018) used ordered oxygen complexes to replace O atoms in the gap enhancement, changed the dislocation shear mode from planar slip to wave slip through ordered gap complexes, and promoted double cross-slip through the formation of Frank-Read sources (a mechanism to explain the generation of multiple dislocations), thus promoting dislocation proliferation, deformation, and stretching to achieve a massive increase in strength and plasticity of TiZrHfNb Bio-HEAs, as shown in Figure 16, the tensile strength is enhanced (by 48.5 ± 1.8 percent), and ductility is substantially improved (by 95.2 ± 8.1 percent). As with metallic elements, small amounts of specific non-metallic elements have a strengthening effect on Bio-HEAs, while transitional doping deteriorates the strengthening effect.

Processing is another crucial factor affecting the strength and ductility of the alloy under the premise that the compositions of the Bio-HEAs group elements are clearly defined. Common preparation methods for Bio-HEAs include vacuum arc melting (Chen et al., 2016), electromagnetic induction melting (George et al., 2020), powder metallurgy techniques (Hu et al., 2022), magnetron sputtering (Dvurečenskij et al., 2022), laser electron beam melting (Arif et al., 2022), and 3D printing additive manufacturing (Wang S. et al., 2022). Smelting is the most common preparation method for Bio-HEA, followed by powder metallurgy and additive manufacturing (Arif et al., 2021). The preparation of homogeneous bulk bioethanol by conventional arc melting is challenging, it requires several post-processing (such as remelting), which is a complex process and only yields samples of simple shapes (Cieslak et al., 2019; Güler et al., 2022). As a fabrication process that overcomes the limitations of traditional machining methods, additive manufacturing (AM) is considered as an advanced machining method for generating low-defect HEA specimens, which has the advantage of producing parts with complex geometries with high precision and achieving large-scale customization to maximize material, energy and time savings (Cui et al., 2021; Du et al., 2021). Recently, AM techniques commonly used for Bio-HEA preparation include directed energy deposition (DED) and powder bed fusion (PBF) (Lin Y. C. et al., 2021; Hassan et al., 2021). Directed energy deposition (DED) is an advanced preparation method based on powder feeding technology that allows higher precision shape customization. The advantages of DED are material design, preparation of different types of coatings and fast repair (Yadav et al., 2021; Yang F. et al., 2022). In contrast, selective laser melting (SLM) and selective electron beam melting (SEBM) offer higher processing accuracy and minimal surface roughness. In addition, in PBF technology, due to the extremely high cooling rate (10^5^–10^7^ K/s), smaller microstructures can be obtained compared to the conventional casting process (100 K/s), which greatly improves the mechanical properties of the part (Huang et al., 2021; Muftah et al., 2021; Ocak and Goller, 2021). For HEAs, the high cooling rate can greatly inhibit the segregation of elements and make it easier to achieve a solid solution structure with a strengthening effect (Zhu et al., 2021).

A change from one preparation technique to another can dramatically affect strength and ductility (Feng et al., 2021, Feng et al., 2022 J.; Wei et al., 2022b). Even for the same technology, the influence of the processing route on the product forming has to be taken into account. Adjusting the processing time and the processing temperature can cause changes in the microstructure and internal defects of the alloy. In addition, the as-prepared alloy samples may have undergone extensive post-treatment processes, such as homogenization, forging, cold and hot rolling, annealing, tempering, aging, etc. This complex process situation, coupled with the elemental diversity of Bio-HEAs, makes it nearly impossible to quantify the effect of processing on the strength and ductility of the alloy, and more precise characterization and analytical tools are needed to address this issue.

The microstructure that most directly affects the mechanical properties of Bio-HEAs also has various forms, including phase composition, grain size, dislocation density, twinning, layer dislocations, size and distribution of precipitated phases, etc. The main phase structures in Bio-HEAs mentioned earlier include BCC, FCC, HCP, Laves, B2, and IC, and MB, MC, MN, MO, and MSi formed by doping with non-metallic elements, as shown in Figure 7. Most single-phase Bio-HEAs are BCC structures, where the elements randomly occupy lattice positions and start BCC phases under the effect of high entropy. The atomic packing density of the BCC phase is 68%, smaller than that of the FCC phase (74%) and the HCP phase (74%). The spatial structure of the BCC phase structure has a higher gap, which makes it easier for small radius solute atoms to enter between the BCC lattice to form interstitial reinforcements, such as the non-metallic elements mentioned previously, without changing the phase structure. Dual solid solution phases Bio-HEAs involve BCC1+BCC2, BCC + HCP, BCC + FCC, BCC + Laves, BCC + B2, and BCC + TC, usually with the B2 phase or BCC phase as the matrix, and the second phase is induced in the alloy by composition adjustment or heat treatment. The multi-phase structure of Bio-HEAs still mainly uses a single-phase or a double phase as the dominant phase and the rest of the phases as the precipitating phases, which play a role in regulating the strength and ductility of Bio-HEAs. To date, there have been many studies on reconciling the strength and ductility of HEAs, including inducing transformation-induced plasticity (TRIP) by stressing (Radi et al., 2022), and creating specially tailored eutectic structures (Ostovari Moghaddam and Trofimov, 2021), introducing interstitially ordered oxygen complexes, and producing nanoscale precipitates or lamellar structures.

Even though Bio-HEAs have a large scope for compositional tuning, it is still difficult to design materials with both high strength and good ductility. At the same time, a large number of researchers have also worked on the strength-ductility conflict, for example, in the case of sacrificing a small amount of strength, the ductility can be greatly improved, or a certain ductility can be guaranteed so that the strength of the alloy is greatly enhanced. Various selectable alloying elements, sophisticated processing methods, and microstructure design allow Bio-HEAs to have more strength-ductility options to design specific alloys according to actual needs (Sheikh et al., 2016).

### Elastic properties

Elastic properties characterize the reversible stress-strain of material when sufficient load is applied (Lv et al., 2022). These parameters are essential for analyzing complex mechanical properties (e.g., ductile-brittle behavior), and studying the elastic properties of Bio-HEAs is necessary because they open up an almost unlimited compositional space in material design. And as a new medical alloy material, elastic properties are also an essential factor in determining the application prospects of Bio-HEAs.

Metals and alloys have a long history of use as bone implants. Still, implants made of traditional medical metal material types are usually much harder than natural bone, leading to stress shielding effects, which is the primary factor in bone resorption and eventual failure of such implants (Wang et al., 2016). The stress shielding effect refers to the fact that when metal implants are aligned parallel to human bone, the modulus of the metal is often more than ten times that of the bone. The huge difference in modulus makes the stresses mainly borne by the alloy. At the same time, the human bone is left unstressed, or under less stress for a long period, bone regeneration is inhibited, and bone atrophy occurs, which leads to implant failure. The modulus of elasticity of human cortical bone (dense bone) is 3–30 Gpa, while the modulus of elasticity of bone trabeculae (cancellous bone) is even lower at 0.02–2 GPa (Wu et al., 2018). Most current metallic materials for implants have a much higher modulus than bone. For example, Ti-6Al-4V has a modulus of about 110 Gpa, while CoCrMo alloys have a modulus of 210 GPa (Che Ghani et al., 2020) and Table 3 shows the modulus of elasticity of various biomedical alloys compared to bone. Therefore, new materials with low Young’s modulus and other bone-adapted mechanical properties need to be developed to avoid stress shielding at the bone-implant interface.

**TABLE 3:** Mechanical properties of human bone, traditional biomaterials, and Bio-HEAs.

Classification	Materials	Young’s modulus (GPa)	σ_y_ (MPa)	Phase	σ_y_/ ρ (MPa*cm^3^*g^−1^)	Ref
Human bone	Cortical bone	15–30	30–70			Wu et al. (2018)
	Cancellous bone	0.01–1.57	4–12			Uppal et al. (2022)
Traditional biomaterials	Co-Cr alloys	210–253	448–1,606			Moghadasi et al. (2022)
	Stainless steel	210–253	221–1,213			Moghadasi et al. (2022)
	Pure Ti	110	485			Moghadasi et al. (2022)
	Ti-6Al-4V	116	896–1,034			Moghadasi et al. (2022)
	Mg alloys	30–50	100–235			Moghadasi et al. (2022)
Bio-HEAs	Hf_0.4_Nb_1.54_Ta_1.54_Ti_0.89_Zr_0.64_	125.0	822	BCC	79.1	Feuerbacher et al. (2015)
	Hf_0.5_Mo_0.5_NbTiZr	123.1	1,150	BCC	149.4	Guo et al. (2016)
	HfMo_0.25_NbTaTiZr	121.0	1,112	BCC	112.2	Juan et al. (2016)
	HfMo_0.5_NbTaTiZr	131.9	1,260	BCC	140.4	Liu et al. (2017)
	HfMoNbTaTiZr	147.0	1,512	BCC	152.1	Juan et al. (2015)
	HfMoNbTiZr	139.2	1,575	BCC	181.3	Guo et al. (2015)
	HfNbTaTiZr	110.6	1,073	BCC	108.4	Lin et al. (2015)
	Mo_0.1_NbTiV_0.3_Zr	106	932	BCC	141.2	Huang et al. (2017)
	MoNbTaTiW	229.4	1,455	BCC	123.8	Han et al. (2018)
	MoNbTaVW	231.8	1,246	BCC	100.7	Senkov et al. (2011)
	MoNbTaW	257.8	996	BCC	72.9	Han et al. (2018)
	MoNbTiV_0.25_Zr	152.9	1750	BCC	241.2	Zhang et al. (2012)
	MoTaTiV	189.8	1,221	BCC	127.4	Qiao et al. (2017)
	NbTaTiV	133.8	965	BCC	105.2	Qiao et al. (2017)
	NbTiVZr	104.3	1,104	BCC	170.9	Wu et al. (2015)

(Where σ_y_ is the yield strength, σ_y_/ ρ represents the specific strength of the Bio-HEAs).

A high-priority goal in designing new metallic materials for load-bearing implant applications is to reduce Young’s modulus in the major loading direction approximating that of cortical bone. In recent years, some biological HEAs and MEAs have been developed (Wang et al., 2017; Wang L. et al., 2018), and the elastic moduli of TiZrHf (HCP), TiZrNbHfTa (BCC), TiZrNbHf (BCC), and TiZrTaHf (BCC) alloys are 111, 103, 83, and 86GPa, respectively (Hu et al., 2021). The elastic modulus of these alloys is relatively low, about half that of CoCr alloys and stainless steel, which is closer to human bone and can effectively reduce the effect of stress shielding.

To further verify the advantages of elastic properties, in addition to the research on Young’s modulus, the current research on Bio-HEAs involves single-crystal elastic constant, polycrystalline elastic modulus, and Debye temperature (Huang and Vitos, 2022). The single-crystal elastic constant C_ij_ describes the response of a material to an external load and can be determined from the stress-strain and or energy-strain relationships (Chanda et al., 2021). The single-crystal elastic constants and polycrystalline elastic moduli of Bio-HEAs can be studied experimentally (*in situ* neutron diffraction, tensile tests, and ultrasonic resonance frequency techniques) and computationally (first-principles calculations) (Diao et al., 2017). For Bio-HEAs cubic crystals, there are three fundamental, independent modes of elastic deformation, namely expansion due to hydrostatic stress, shearing along the cubic crystal axis on the cubic plane of the crystal, and the shear resulting from the plane rotated 45° about the cube axis from the cube face is sheared in a direction perpendicular to that axis. The single-crystal elastic constants describe the relationship between the measured h, k, and l-specific elastic lattice strains, using neutron diffraction and the macroscopic stresses applied to the polycrystalline HEA material. The polycrystalline elastic modulus is calculated by the Voigt–Reuss–Hill method based on the single-crystal elastic coefficients to obtain Young’s modulus E, bulk modulus B, shear modulus G, microhardness H, Poisson’s ratio υ, and Cauchy pressure of Bio-HEAs, and thus further evaluate the mechanical properties of Bio-HEAs such as strength, hardness, and elastic anisotropy.

**FIGURE 17:**
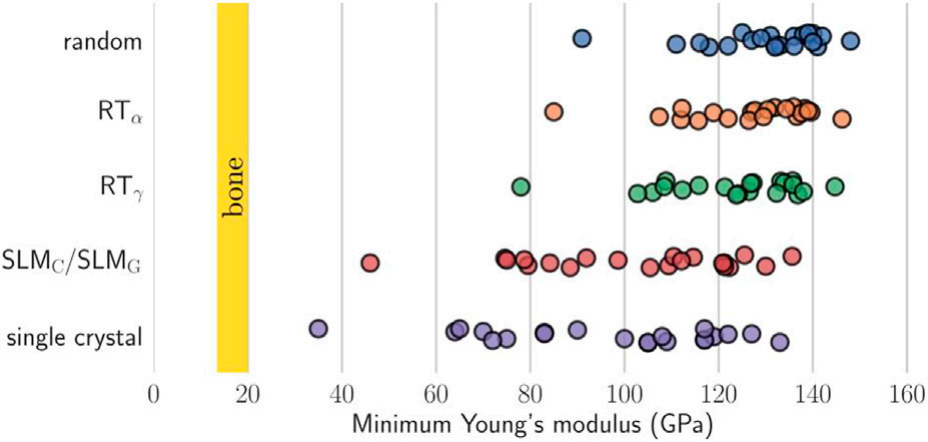
Higher single crystal elastic anisotropy for the present HEAs typically implies a lower minimum Young’s modulus. For comparison, the vertical yellow bars indicate the targeted Young’s modulus of cortical bone (Schönecker et al., 2022).

The elastic anisotropy is related to the formation of microcracks in the material and impacts the mechanical durability of Bio-HEAs. The study of the elastic anisotropy of Bio-HEAs was examined theoretically and experimentally (Duesbery and Vitek, 1998; Fazakas et al., 2014; Tian et al., 2014; Dirras et al., 2016; Lilensten et al., 2018; Laplanche et al., 2019; Akdim et al., 2021; Raturi et al., 2022). Virtual crystal approximation (VCA), coherent potential approximation (CPA), and special quasi-random structure (SQS) simulations by calculating the BCC HEA with elastic stiffness constants (Fazakas et al., 2014; Tian et al., 2014). For example, Tian et al. (Tian et al., 2014) showed that molybdenum-containing alloys are almost isotropic compared to molybdenum-free alloys of the TiZrNbMoV_x_ system. By studying the single-crystal elastic properties of 21 Bio-HEAs, as shown in Figure 17, Schonecher et al. (Schönecker et al., 2022) verified that the valence electron number has a dominant effect on the elastic anisotropy and crystal orientation of low Young’s modulus and high torsional modulus in HEAs, and discussed the potential of using single crystals or woven aggregates to reduce Young’s modulus in Bio-HEAs.

### Wear resistance

When conventional biomedical alloys are implanted, friction and wear are usually accelerated due to the corrosive physiological environment, resulting in considerable corrosion and wear of the material, severely reducing the service period (Chiba et al., 2007). Therefore, biomedical metal implant alloys need excellent biocompatibility, high corrosion, and good wear resistance. However, due to the low wear resistance of Ti-based alloys, particle diseases resulting from the wear debris may occur when they are performing in physiological environments (Long and Rack, 1998; Geetha et al., 2009). Ti-Zr-Nb-based refractory HEAs typically exhibit a body-centered cubic (BCC) solid solution structure and thus have higher hardness, yield strength, and wear resistance than Ti-6Al-4V, making them highly resistant to plastic deformation and fracture under high loads.

**FIGURE 18:**
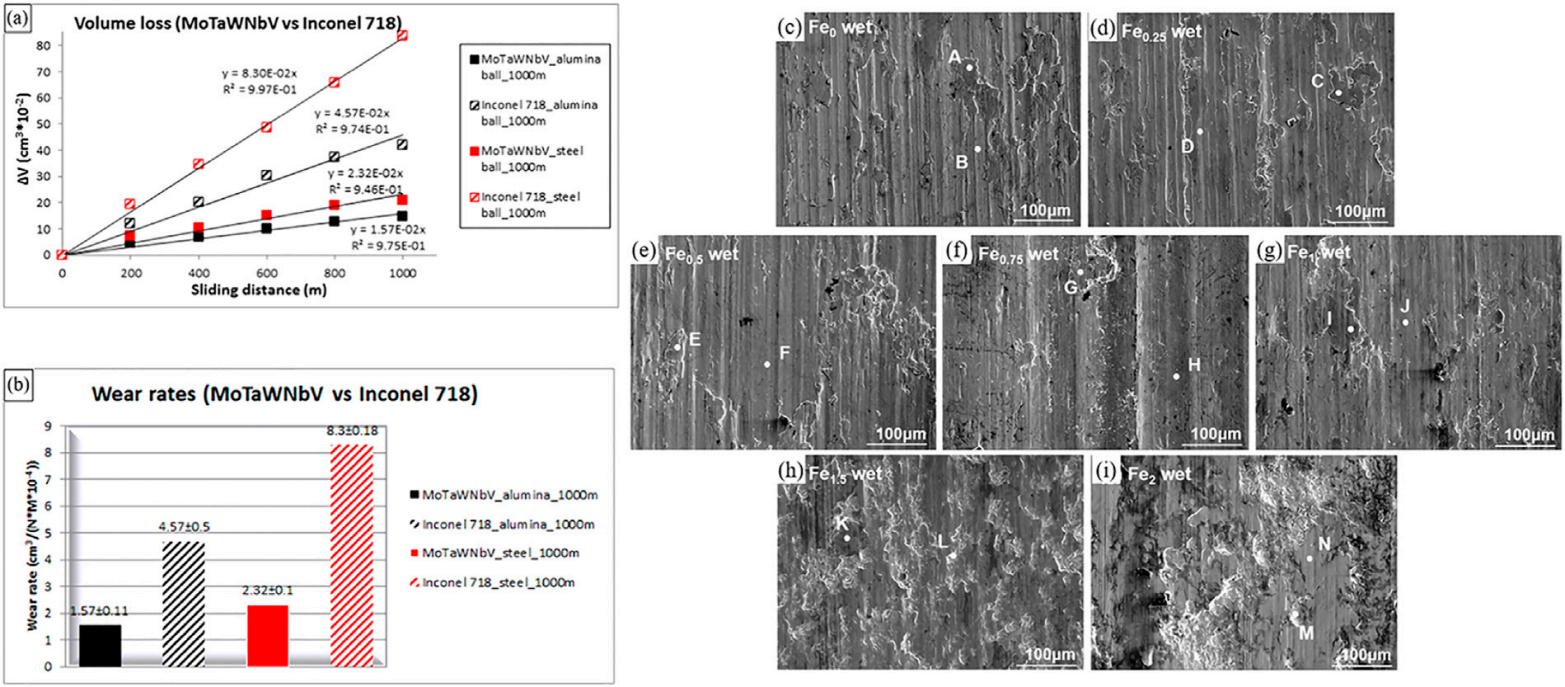
**(A, B)** Comparison of frictional properties of MoTaWNbV high entropy alloy and Inconel 718 conventional high-temperature alloy, using aluminum oxide and steel balls as counter body materials (Poulia et al., 2016). **C–I** SEM images of worn-scar surfaces of the Ti-Zr-Hf-Nb-Fe_x_ HEAs under PBS wet wear conditions (Wang et al., 2022c).

The wear resistance of Bio-HEAs is the key to their service life (Moazzen et al., 2022; Zhang et al., 2022). Poulia et al. (Poulia et al., 2016) analyzed the influence mechanism of the solid solution phase on relative wear resistance. They found that MoTaWNbV high-entropy alloy with a single-phase BCC structure could effectively absorb the energy during wear due to the abundant slip system in the BCC structure of the alloy, the BCC structure of this alloy possesses more slip systems. However, MoTaNbZrTi high-entropy alloy with a dual-phase BCC + HCP structure will form an oxide on the surface during wear to lubricate the surface and improve wear resistance. Both alloys have higher wear resistance than conventional high-temperature alloys. Figure 18 shows the wear resistance of MoTaWNbV and MoTaNbZrTi alloys compared to conventional high temperature alloys. To further investigate the wear resistance of Bio-HEAs under physiological environments, some researchers used PBS solutions to test the wear resistance of TiZrHfNbFe_x_ HEAs under real service conditions (Wang W. et al., 2022). Figure 18 shows the fatigue wear of Bio-HEAs under applied load, where the deformed layer of the alloy surface is stripped off, and the exposed fresh metal surface reacts with the PBS solution and exhibits better resistance to wet wear than Ti-6Al-4V. In addition, the content of Fe elements was found to affect the wear mechanism of these Bio-HEAs, from abrasive and corrosive wear of Fe_0_ to Fe_1_ alloy to fatigue and corrosion wear of Fe_1.5_ and Fe_2_ alloys after sliding in PBS solution.

## Conclusion and outlook

In conclusion, Bio-HEAs have potential applications in the biomedical field but are still in the laboratory stage. The immature preparation process, high cost, and clinical experiments have not yet been realized, making it difficult for Bio-HEAs to be widely used. The design theory, mechanical properties, and biocompatibility of Bio-HEAs are summarized, and their future directions are partially discussed as follows:

(1). There are currently relatively systematic design theories for HEAs, including semi-empirical guidelines for characteristic quantities such as mixing entropy, valence electron concentration, atomic radius difference, mixing enthalpy, and electronegativity. However, a great deal of validation work is still required for Bio-HEAs to design novel alloys. A simulation is an essential approach for the composition design of Bio-HEAs to predict material properties from electronic and atomic scales. Combined with machine learning, it can significantly reduce the time and cost of designing Bio-HEAs with new compositions.
(2). The phase structure of Bio-HEAs is dominated by the single phase of BCC, and the second phases of multi-phase Bio-HEAs include FCC, HCP, Laves, B2, and IC phases. Still, not all of these precipitated phases exhibit beneficial effects on the alloy, and excessive doping will destroy the continuity of the matrix. It is necessary to verify the reinforcement mechanism of precipitated relative Bio-HEAs through continuous exploratory experiments to achieve the goal of multiphase synergistic reinforcement.
(3). In the biomedical field, the application trend of Bio-HEAs is biased towards metal implants, which requires rigorous experimental to demonstrate the biosafety and service life of Bio-HEAs, and the continuous efforts of several research teams.
