# Corrigendum: Bio-high entropy alloys: Progress, challenges, and opportunities

**DOI:** 10.3389/fbioe.2022.1091752

**Published:** 2022-11-18

**Authors:** Junyi Feng, Yujin Tang, Jia Liu, Peilei Zhang, Changxi Liu, Liqiang Wang

**Affiliations:** ^1^ School of Materials Engineering, Shanghai University of Engineering Science, Shanghai, China; ^2^ Affiliated Hospital of Youjiang Medical University for Nationalities, Baise, China; ^3^ State Key Laboratory of Metal Matrix Composites, School of Materials Science and Engineering, Shanghai Jiao Tong University, Shanghai, China

**Keywords:** biological high-entropy, compositon design, mechanical properties, implant, biocompatibility

In the published article, Affiliations 2 and 3 were switched. Affiliation 2 should be “Affiliated Hospital of Youjiang Medical University for Nationalities, Baise, China,” whereas Affiliation 3 should be “State Key Laboratory of Metal Matrix Composites, School of Materials Science and Engineering, Shanghai Jiao Tong University, Shanghai, China.”

The authors apologize for this error and state that this does not change the scientific conclusions of the article in any way. The original article has been updated.

## Publisher’s note

All claims expressed in this article are solely those of the authors and do not necessarily represent those of their affiliated organizations, or those of the publisher, the editors and the reviewers. Any product that may be evaluated in this article, or claim that may be made by its manufacturer, is not guaranteed or endorsed by the publisher.

